# A meta-analysis of associations of *LEPR* Q223R and K109R polymorphisms with Type 2 diabetes risk

**DOI:** 10.1371/journal.pone.0189366

**Published:** 2018-01-02

**Authors:** Yunzhong Yang, Tianhua Niu

**Affiliations:** 1 Department of Global Biostatistics and Data Science, Tulane University School of Public Health and Tropical Medicine, New Orleans, LA, United States of America; 2 Department of Biochemistry and Molecular Biology, Tulane University School Medicine, New Orleans, LA, United States of America; ENEA Centro Ricerche Casaccia, ITALY

## Abstract

**Background:**

Leptin receptor (LEPR) plays a pivotal role in the control of body weight, energy metabolism, and insulin sensitivity. Various genetic association studies were performed to evaluate associations of *LEPR* genetic variants with type 2 diabetes (T2D) susceptibility.

**Methods:**

A comprehensive search was conducted to identify all eligible case-control studies for examining the associations of *LEPR* single nucleotide polymorphisms (SNPs) Q223R (rs1137101) and K109R (rs1137100) with T2D risk. Odds ratios (OR) and corresponding 95% confidence intervals (CIs) were used to measure the magnitudes of association.

**Results:**

For Q223R, 13 studies (11 articles) consisting of a total of 4030 cases and 2844 controls, and for K109R 7 studies (7 articles) consisting of 3319 cases and 2465 controls were available. Under an allele model, Q223R was not significantly associated with T2D risk (OR = 1.09, 95% CI: 0.80–1.48, P-value = 0.5989), which was consistent with results obtained under four genotypic models (ranges: ORs 1.08–1.20, 95% CIs: 0.58–2.02 to 0.64–2.26; P-values, 0.3650–0.8177, which all exceeded multiplicity-adjusted α = 0.05/5 = 0.01). In addition, no significant association was found between K109R and T2D risk based on either an allele model (OR = 0.93, 95% CI: 0.85–1.03, P-value = 0.1868) or four genotypic models (ranges: ORs 0.81–0.99, 95% CIs: 0.67–0.86 to 0.97–1.26, P-values, 0.0207–0.8804 which all exceeded multiplicity-adjusted α of 0.01). The magnitudes of association for these two SNPs were not dramatically changed in subgroup analyses by ethnicity or sensitivity analyses. Funnel plot inspections as well as Begg and Mazumdar adjusted rank correlation test and Egger linear regression test did not reveal significant publication biases in main and subgroup analyses. Bioinformatics analysis predicted that both missense SNPs were functionally neutral and benign.

**Conclusions:**

The present meta-analysis did not detect significant genetic associations between *LEPR* Q223R and K109R polymorphisms and T2D risk.

## Introduction

Type 2 diabetes (T2D), a metabolic disorder that is characterized by hyperglycemia (i.e., high blood glucose) in the context of insulin resistance and a relative lack of insulin, is the most common form of diabetes, accounting for at least 90% of diabetic individuals globally [1]. Recent studies suggest that T2D is increasing rapidly worldwide [2]. The development of T2D is multifactorial, which involves both environmental factors and genetic variants [3].

Leptin (LEP, also called OB for obese) is an adipocyte-derived hormone produced mainly by white adipose tissue, which regulates appetite, energy metabolism, body weight, and insulin sensitivity [4–6]. The word “leptin”, which is from the Greek word ‘leptos’, means ‘thin’, referring to its regulating functions on appetite, food intake and energy homeostasis. LEP exerts its important physiological effect on the regulation of fat metabolism by binding to LEP receptor (LEPR, also called CD295 and OBR) [6–8], which is a single transmembrane protein that belongs to class I cytokine receptor family distributed in a variety of tissue types [9]. Both *LEP* and *LEPR* genes have been cloned in humans [10, 11], and have been mapped to chromosome regions 7q32.1 [12] and 1p31.3 [13, 14], respectively.

The LEPR protein has six isoforms designated OBRa, OBRb, OBRc, OBRd, OBRe, and OBRf, which are obtained by alternative splicing [15]. Although all six isoforms share an identical extracellular domain [16], only OBRb (i.e., the long full-length isoform) contains intracellular motifs required for the transduction of intracellular signaling [17, 18]. Of them, OBRb is considered to be the major isoform involved in appetite control [19], which is primarily expressed in hypothalamic regions [16]. Nevertheless, OBRb is found to be expressed in pancreatic islets, mediating the inhibitory effects of LEP on insulin secretion [20]. Upon LEP binding to OBRb, an OBRb/Janus kinase 2 (JAK2) complex is formed, resulting in cross-phosphorylation. The tyrosine residue, Tyr1138 on OBRb, is important for signal transducer and activator of transcription 3 (STAT3) activation, which activates suppressor of cytokine signaling 3 (SOCS3) expression. This leads to a negative inhibition of LEP signaling through Tyr985 and additional sites on JAK2. Mitogen-activated protein kinase (MAPK) and insulin receptor substrate/phosphatidyl-inositol 3’ kinase (PI3K) pathways can also be activated following JAK2 phosphorylation [21]. Through binding to OBRb, LEP can activate multiple signal transduction pathways and particularly the JAK2/STAT3 pathway for controlling food intake and energy balance.

**Fig 1:**
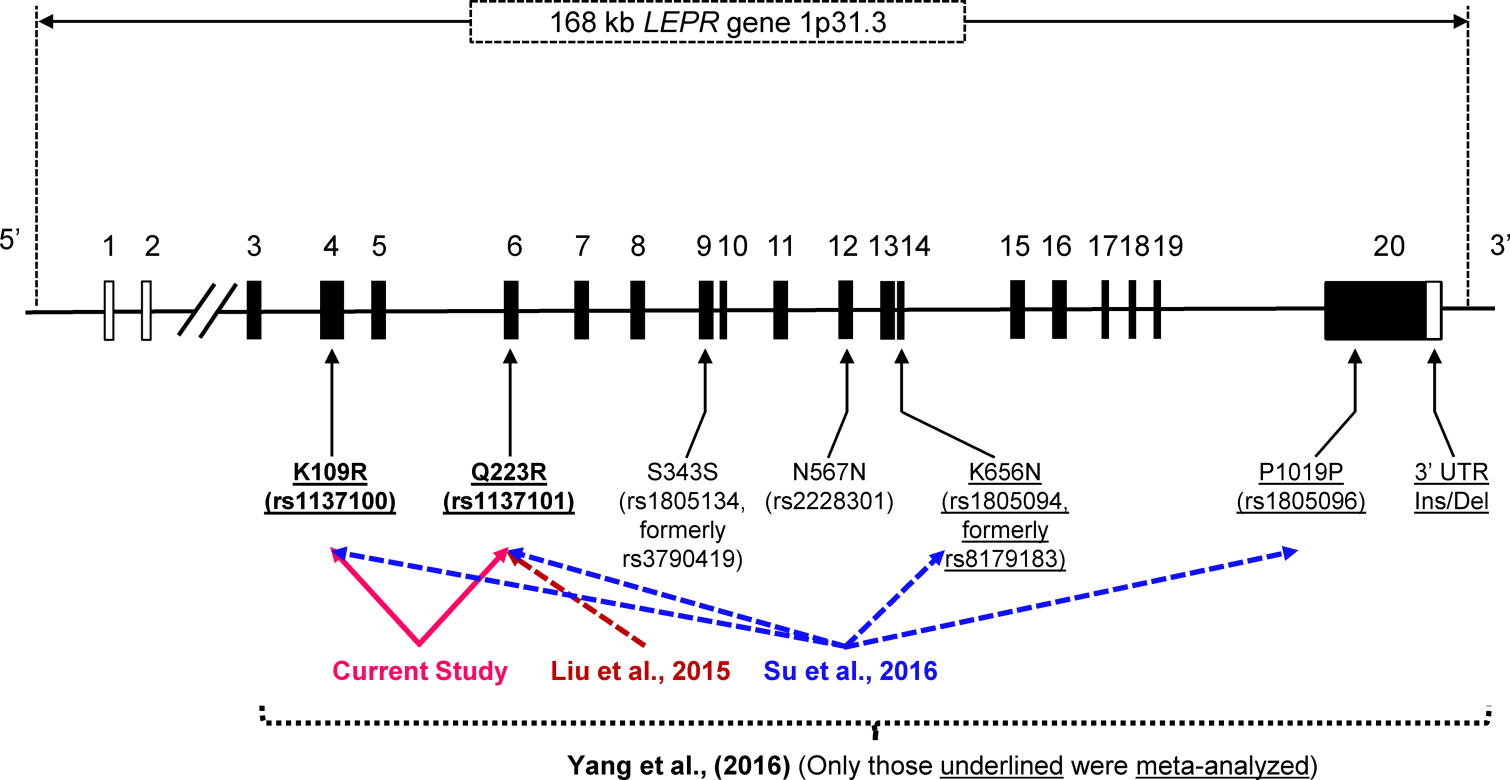
A schematic diagram of *LEPR* exon-intron gene structure spanning 168-kilobase (kb) displaying genomic locations of *LEPR* K109R (rs1137100) (exon 4), Q223R (rs1137101) (exon 6), S343S (rs1805134, formerly rs3790419) (exon 9), N567N (rs2228301) (exon 12), K656N (rs1805094, formerly rs8179183) (exon 14), P1019P (rs1805096) (exon 20), and 3’ untranslated region (UTR) Ins/Del polymorphisms (exon 20) based on gene structures shown in Thompson et al. (1997) [81] and Hansel et al. (2009) [82], with applications of SeqVISTA [83, 84] to map the locations of these genetic variants. Only Q223R, K109R, K656N, P1019P and 3’ UTR Ins/Del (i.e., underlined) polymorphisms were meta-analyzed by Yang et al. (2016) [24]. Only Q223R was meta-analyzed by Liu et al. (2015) [69], and only Q223R, K109R, K656N, and P1019P were meta-analyzed by Su et al. (2016) [70]. Filled boxes indicate protein-coding regions, and open boxes indicate non-protein-coding regions, i.e., UTRs. Abbreviations: Del deletion; Ins, insertion; UTR, untranslated region. Unfilled boxes are non-coding regions. Not drawn to scale.

To evaluate the potential roles of *LEPR*’s molecular variants in T2D risk, several individual genetic association studies have been conducted by different research groups on polymorphisms located in this gene in different ethnic populations. However, results of these studies are controversial and inconclusive (e.g., for Q223R, studies of [22] and [23] showed effects in opposite directions). Seven *LEPR* genetic polymorphisms, i.e., K109R (rs1137100), Q223R (rs1137101), S343S (rs1805134, formerly rs3790419), N567N (rs2228301), K656N (rs1805094, formerly rs8179183), P1019P (rs1805096), and 3’ untranslated region (UTR) Ins/Del polymorphisms have been previously studied for their associations with T2D risk [24] (Fig 1), however, only two missense single nucleotide polymorphisms (SNPs)—Q223R (rs1137101) and K109R (rs1137100) located in exons 6 and 4 respectively, were most widely examined with regard to their roles in T2D risk, for which sufficient numbers of single studies (i.e., > 5) were obtained for each SNP. We therefore conducted a comprehensive meta-analysis focusing exclusively on these two missense SNPs aiming at elucidating their associations with T2D susceptibility.

## Materials and methods

### Search strategy

We searched relevant studies from the following electronic databases: PubMed, Excerpta Medica Database (EMBASE), Cochrane Library, and Google Scholar up to February 1, 2016. The following search terms were used in the electronic searches: “leptin receptor”, “gene”, “lepr”, “T2D”, “T2D and Type 2 Diabetes” with language restrictions to either English or Chinese. This study was performed in accordance with the Preferred Reporting Items for Systematic Reviews and Meta-Analyses (PRISMA) statement checklist (S1 PRISMA Checklist) and the Meta-analysis of Genetic Association Studies checklist (S2 Checklist).

### Study selection

The inclusion criteria were: (1) an original human-based case-control study using either a hospital-based or a population-based design; (2) a clear definition of T2D; (3) the relationship between either Q223R (rs1137101) or K109R (rs1137100) and T2D risk was evaluated; and (4) providing sufficient data for calculating genotype and allele odds ratios (ORs) with their respective corresponding 95% confidence intervals (CIs). The exclusion criteria were: (1) reviews, conference abstracts, editorials and letters, (2) animal and in vitro studies, and (3) data about genotype frequencies could not be obtained. In case of overlapping or repeated studies, the one with most completed information was chosen. In addition, if more than one study shared the same subjects, the one with smaller sample size is excluded. All assessments were performed independently by two reviewers (YY and TN).

### Data extraction

Data extraction was performed independently by two investigators (YY and TN) based on a pre-defined standard protocols. Any disagreements were solved by discussion. From each qualified study, the following information was collected: year of publication, first author’s name, study location, ethnicity, source of controls (population-based or hospital-based), diagnosis criteria of T2D (i.e., how T2D is defined), sample sizes and respective genotypic frequencies in case and control groups, mean±standard deviation (SD) of age, distribution of gender, genotyping methods, and Hardy-Weinberg equilibrium (HWE) in controls (To present study characteristics more succinctly, T2D diagnosis criteria, genotyping methods, and HWE in controls were omitted from Tables 1 and 2). For each variable, corresponding measurements were shown using the same unit.

**Table 1:** General characteristics of 13 included studies for *LEPR* Q223R*.

								Age (Mean±SD)		Gender (M/F)		
First author, Year	Ethnicity	Definition of T2D	Source of controls	# Cases	Genotype Freq. in cases (AA/AG/GG)	# Controls	Genotype Freq. in controls (AA/AG/GG)	Case	Control	Case	Control	NOS
Ali Etemad, 2013 (Malay) [49]	Malay	IDF	PB	145	42/17/86**	133	22/20/91**					
Ali Etemad, 2013 (Chinese) [49]	Chinese	IDF	PB	49	13/0/36**	71	6/5/60**	61.9±9.8**	53.3±12.4**	191/93**	158/123**	7
Ali Etemad, 2013 (Indian) [49]	Indian	IDF	PB	90	37/7/46**	77	23/15/39**					
Bo Jiang, 2014 [50]	Chinese	WHO	PB	8	4/65/273	176	3/33/117	68.1±6.4***	67.1±7.1	121/246	75/101	9
Ghorban Mohammadzadeh, 2013 [51]	Iranian	ADA	HB	144	5/59/80	147	5/62/80	54.33±8.85	52.53±7.31	58/86	63/84	8
Malgorzata Roszkowska-Gancarz, 2014 [52]	Polish	NA	NA	190****	48/98/44****	542****	147/266/129****	47.2±5.3	NA	70/120****	127/225****	7
W-L Liao, 2012 [23]	Taiwanese	ADA	NA	999	8/194/796	45	1/8/36	NA	NA	489/510	NA	7
Kyong Soo Park, 2006 [53]	Korean	ADA	HB	775	11/177/578	688	13/148/523	58.9±10.5	64.2±4.2	361/414	308/380	8
R-T Gan, 2012 [22]	Chinese	NA	PB	301	18/83/200	172	4/47/121	52.67±10.74***	52.8±7.98	NA	NA	8
Lin-Shuang Zhao, 2008a [54]	Chinese	WHO	NA	436	85/156/195	160	91/30/39	NA	51.1±2.2	272/164	91/69	8
Devi Murugesan, 2010 [55]	Indian	NA	HB	150	30/67/53	150	73/55/22	NA	NA	NA	NA	7
Yangdan Zhang, 2011 [56]	Chinese	WHO	HB	172	4/40/128	164	1/63/100	66.52±12.94	64.7±14.8	90/82	87/77	8
Hong Sun, 2011 [57]	Chinese	WHO	PB	210	2/54/147	319	10/57/239	NA	NA	79/131	181/138	9

*For *LEPR* Q223R, a total of 13 studies from 11 articles were included. In the study of Etemad et al. (2013) [49] three studies were included, i.e., Study_1, Maylay; Study_2, Chinese; and Study_3, Indian. Abbreviations: ADA, American Diabetes Association; Freq, frequency; HB, hospital-based; IDF, International Diabetes Federation; NOS, Newcastle-Ottawa scale; PB, population-based; SD, standard deviation; T2D, type 2 diabetes; T2D, type 2 diabetes; WHO, World Health Organization; NA, not available. The number of cases (or controls) may not be equal to the sum of the genotype frequencies because of genotyping missing data.

**Genotype frequencies for cases and controls were calculated from respective percentage data shown in Table 2 of Etemad et al. (2013) [49] for Malays, Chinese, and Indians, which were the same as reported by Yang et al. (2016) [24]. Summary statistics for Age and Gender were obtained for the total study sample combining Malay, Chinese, and Indian subgroups together of Etemad et al. (2013) [49].

***Summary statistics for Age in T2D controls were computed manually based on subgroup data of Jiang et al. (2014) [50], respectively.

****The data of Roszkowska-Gancarz et al. (2014) [52] were based on 542 controls (128 centenarians, 414 young controls), and 190 T2D cases only. Genotype frequencies for cases and controls and were calculated from respective percentage data shown in Table II of Roszkowska-Gancarz et al. (2014) [52].

**Table 2:** General characteristics of 7 included studies for *LEPR* K109R*.

								Age (Mean±SD)		Gender (M/F)		
First author, Year	Ethnicity	Definition of T2D	Source of controls	# Cases	Genotype Freq. in cases (AA/AG/GG)	# Controls	Genotype Freq. in controls (AA/AG/GG)	Case	Control	Case	Control	NOS
Bo Jiang, 2014 [50]	Chinese	WHO	PB	369	3/81/184	176	3/35/72	68.1±6.4**	67.1±7.1	121/246	75/101	9
Malgorzata Roszkowska-Gancarz, 2014 [52]	Polish	NA	NA	190***	48/98/44***	542***	147/266/129***	47.2±5.3	NA**	70/120***	197/345***	7
W-L Liao, 2012 [23]	Taiwanese	ADA	NA	999	23/265/705	80	1/29/50	NA	NA	489/510	NA	7
Kyong Soo Park, 2006 [53]	Korean	ADA	HB	775	31/238/496	688	22/200/461	58.9±10.5	64.2±4.2	361/414	308/380	8
Yanchun Qu, 2007 [58]	Chinese	ADA	PB	317	11/93/213	282	8/71/203	49.3±13.7	45.2±5.7	156/161	170/112	9
Devi Murugesan, 2010 [55]	Indian	NA	HB	150	10/40/100	150	10/48/91	NA	NA	NA	NA	7
Miguel Cruz, 2010 [59]	Mexican	ADA	PB	519	223/204/59	547	204/211/49	53.4±7.4	43.6±6.6	NA	NA	8

*For *LEPR* K109R, a total of 7 studies from 7 articles were included. Abbreviations: ADA, American Diabetes Association; Freq, frequency; HB, hospital-based; IDF, International Diabetes Federation; NOS, Newcastle-Ottawa scale; PB, population-based; SD, standard deviation; T2D, type 2 diabetes; T2D, type 2 diabetes; WHO, World Health Organization; NA, not available.

**Summary statistics for Age in T2D controls were computed manually based on subgroup data of Jiang et al. (2014) [50], respectively.

***The data of Roszkowska-Gancarz et al. (2014) [52] were based on 542 controls (128 centenarians, 414 young controls), and 190 T2D cases only. Genotype frequencies for cases and controls were calculated from respective percentage data shown in Table II of Roszkowska-Gancarz et al. (2014) [52].

### Quality assessment

Two authors (YY and TN) evaluated each individual study’s quality independently according to the Newcastle-Ottawa scale (NOS) [25], which assesses the quality of each individual study in three sections: (1) selection of study subjects: 0–4; (2) comparability of study subjects: 0–2; and (3) clinical outcome: 0–3. The NOS score has a range of 0–9; and a score ≥ 7 is indicative of a good quality, e.g., [26, 27]. Studies with a NOS score ≥ 6 are considered to be of sufficient quality for inclusion in a meta-analysis (e.g., [24, 28]).

### Statistical analysis

The ORs with 95% CIs were computed to evaluate respective associations of *LEPR* Q223R and K109R SNPs with T2D risk. For each polymorphism, 5 genetic models were employed, i.e., (1) an allele model (G vs. A), (2) a homozygote model (GG vs. AA), (3) a heterozygote model (AG vs. AA), (4) a dominant model (GG+AG vs. AA), and (5) a recessive model (GG vs. AG+AA).

Heterogeneity among studies was assessed by Cochrane’s Q-test [29], which follows a chi-square distribution. I^2^ statistic, which is on a scale of 0–100% (0–25%, no heterogeneity; 25–50%, moderate heterogeneity; 50–75%, large heterogeneity; 75–100%, extreme heterogeneity) [30], is also computed. A Cochrane’s Q test P-value < 0.10 [30] or an I^2^ > 50% [31] was considered indicative of a statistically significant heterogeneity. A random effects model (the DerSimonian and Laird method) [32] was employed when a significant heterogeneity was detected among studies. Otherwise, a fixed effects model (the Mantel-Haenszel method) [33] was applied. Subgroup analyses stratified by ethnicity (Chinese populations vs. non-Chinese populations) were performed. The stability of the results was assessed using sensitivity analysis by removing each single study involved in the meta-analysis one at a time to reflect the influence of the individual study to the pooled ORs. The potential presence of publication bias was assessed by means of funnel plot inspection, and both Begg and Mazumdar adjusted rank correlation test [34] and Egger’s linear regression test [35] were applied to test for funnel plot asymmetry. All statistical analyses were conducted using R version 3.2.3 software meta package (https://cran.r-project.org/web/packages/meta/index.html) and metafor package (https://cran.r-project.org/web/packages/metafor/index.html).

### Bioinformatics analysis

A total of 7 *in silico* tools were applied for functional prediction of *LEPR* Q223R and K109R: (1) Mutation Assessor [36] (http://mutationassessor.org), (2) BLOSUM62 [37] (https://www.ncbi.nlm.nih.gov/Class/FieldGuide/BLOSUM62.txt), (3) PROVEAN [38] (http://provean.jcvi.org/index.php), (4) PolyPhen-2 [39] (http://genetics.bwh.harvard.edu/pph2/), (5) PANTHER [40], (6) SNPs&GO [41] (http://snps-and-go.biocomp.unibo.it/snps-and-go/), and (7) SNPs3D [42] (http://www.snps3d.org/). Mutation Assessor [36] calculates a functional impact (FI) score for a protein mutation. A functional impact (FI) score ≤ 0.8, 0.8–1.9, 1.9–3.5 and > 3.5 is indicative of “neutral”, “low impact”, “medium impact”, and “high impact”, respectively [43]. BLOSUM62 is a scoring matrix for amino acid substitutions, such that a negative score is indicative of an evolutionarily less acceptable substitution, and a positive score is indicative of an evolutionarily more acceptable substitution [37]. PROVEAN (Protein Variation Effect Analyzer) computes a PROVEAN score by using a delta alignment score approach [38]. A score ≤ -2.5 and > -2.5 is indicative of “deleterious”, and “neutral”, respectively [44]. PolyPhen-2 [39] computes a Position-Specific Independent Count (PSIC) score ranging from 0 to 1. A criterion used by [44] is that a PSIC score ≤ 0.5 and > 0.5 is indicative of “probably damaging”, and “benign”, respectively. PANTHER [40] computes a substitution position-specific evolutionary conservation (subPSEC). A subPSEC score ≤ -3 (corresponding to a P_deleterious_ ≥ 0.5) and > -3 (corresponding to a P_deleterious_ < 0.5) is indicative of “deleterious” and “neutral”, respectively [45, 46]. A greater P_deleterious_ indicates a tendency to exert more severe impairments on protein function [47]. A SNPs&GO Disease Probability score > 0.5 and ≤ 0.5 is indicative of “deleterious”, and “neutral”, respectively [41]. SNPs3D [42] computes a support vector machine (SVM) score. An SVM score < 0 and ≥ 0 is indicative of “deleterious” and “neutral”, respectively [48].

## Results

### Characteristics of included studies

**Fig 2:**
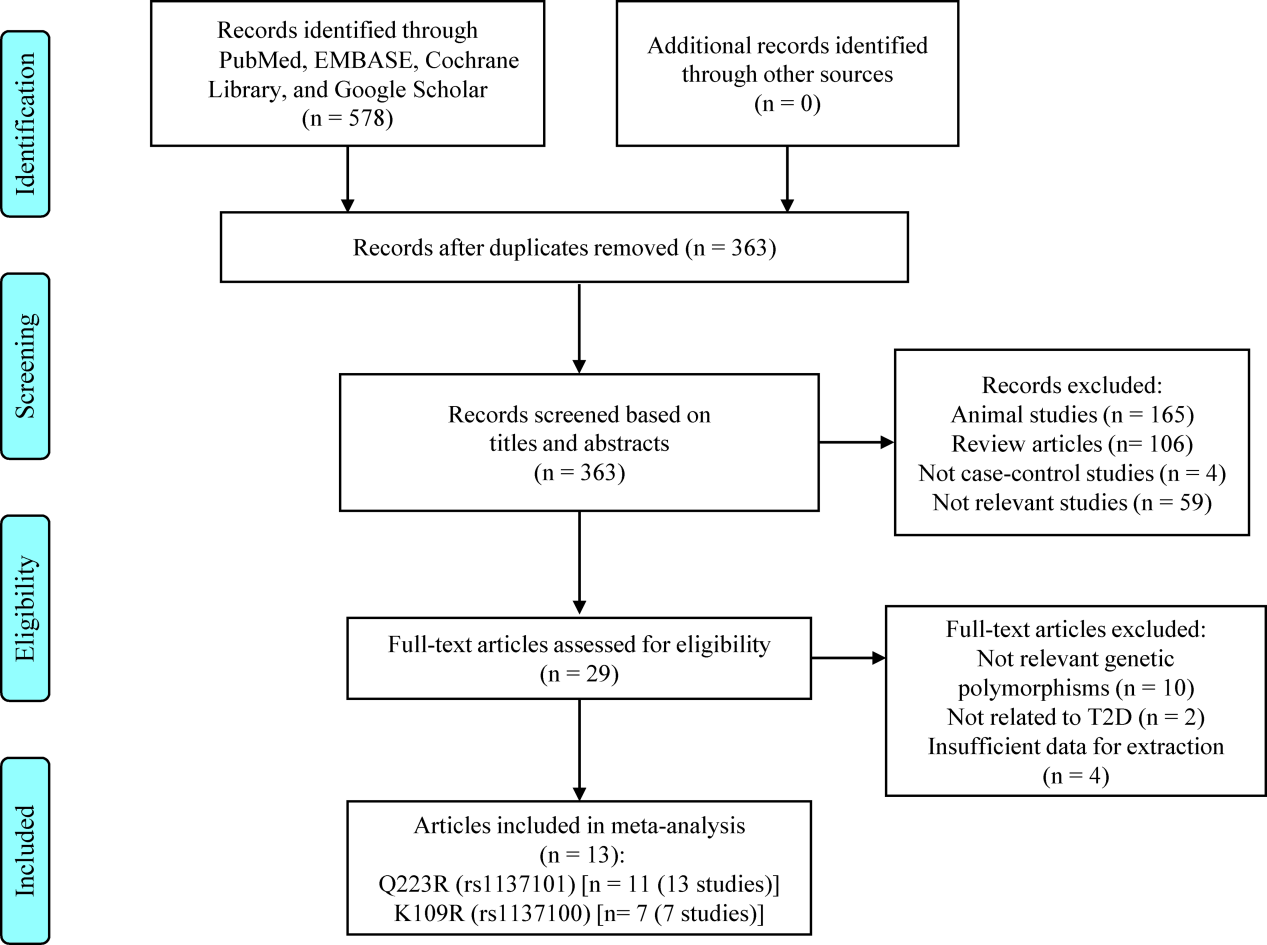
A PRISMA flow diagram depicting the literature search and study selection process. Abbreviations: EMBASE, Excerpta Medica Database; T2D, type 2 diabetes.

A flow diagram depicting the study selection process is shown in Fig 2. An initial literature search identified 578 potentially relevant articles (S3 Electronic Search Strategy and Results). After removing duplicates, there were 363 potentially relevant articles. Based on reviews of titles and abstracts of them, 335 articles were excluded (including 165 animal studies, 106 review articles, 4 articles that are not case-control studies, and 59 studies that were not relevant). Full texts were reviewed for the remaining 29 articles, and 16 of them were further excluded. Finally, 13 articles (10 English articles and 3 Chinese articles) were included in this meta-analysis. For *LEPR* Q223R, 13 studies (7 in Chinese populations and 6 studies in non-Chinese populations) from 11 articles [22, 23, 49–57] were included, comprising 4030 cases and 2844 controls. For *LEPR* K109R, 7 studies (3 in Chinese populations and 4 studies in non-Chinese populations) from 7 articles [23, 50, 52, 53, 55, 58, 59] were included, comprising 3319 cases and 2465 controls. The characteristics of the included studies are presented in Tables 1 and 2 for Q223R and K109R, respectively. The mean±SD for NOS score was 7.82±0.75 (range, 7–9) for Q223R and 7.83±0.89 (range, 7–9) for K109R, respectively. Specifically, for Q223R (variant allele: R223), higher variant allele frequencies (VAFs) were observed in Chinese T2D cases (Mean±SD: 0.82±0.10; range, 0.63–0.89) and controls (Mean±SD: 0.79±0.20; range, 0.34–0.89) than in non-Chinese T2D cases (Mean±SD: 0.64±0.12; range, 0.49–0.80) and controls (Mean±SD: 0.63±0.19; range, 0.33–0.84), respectively (S1 Fig). Further, for K109R (variant allele: R109), higher VAFs were observed in Chinese T2D cases (Mean±SD: 0.83±0.013; range, 0.82–0.84) and controls (Mean±SD: 0.82±0.021; range, 0.81–0.85) than in non-Chinese T2D cases (Mean±SD: 0.40±0.28; range, 0.20–0.80) and controls (Mean±SD: 0.42±0.27; range, 0.23–0.82), respectively (S2 Fig).

### Meta-analysis results

**Fig 3:**
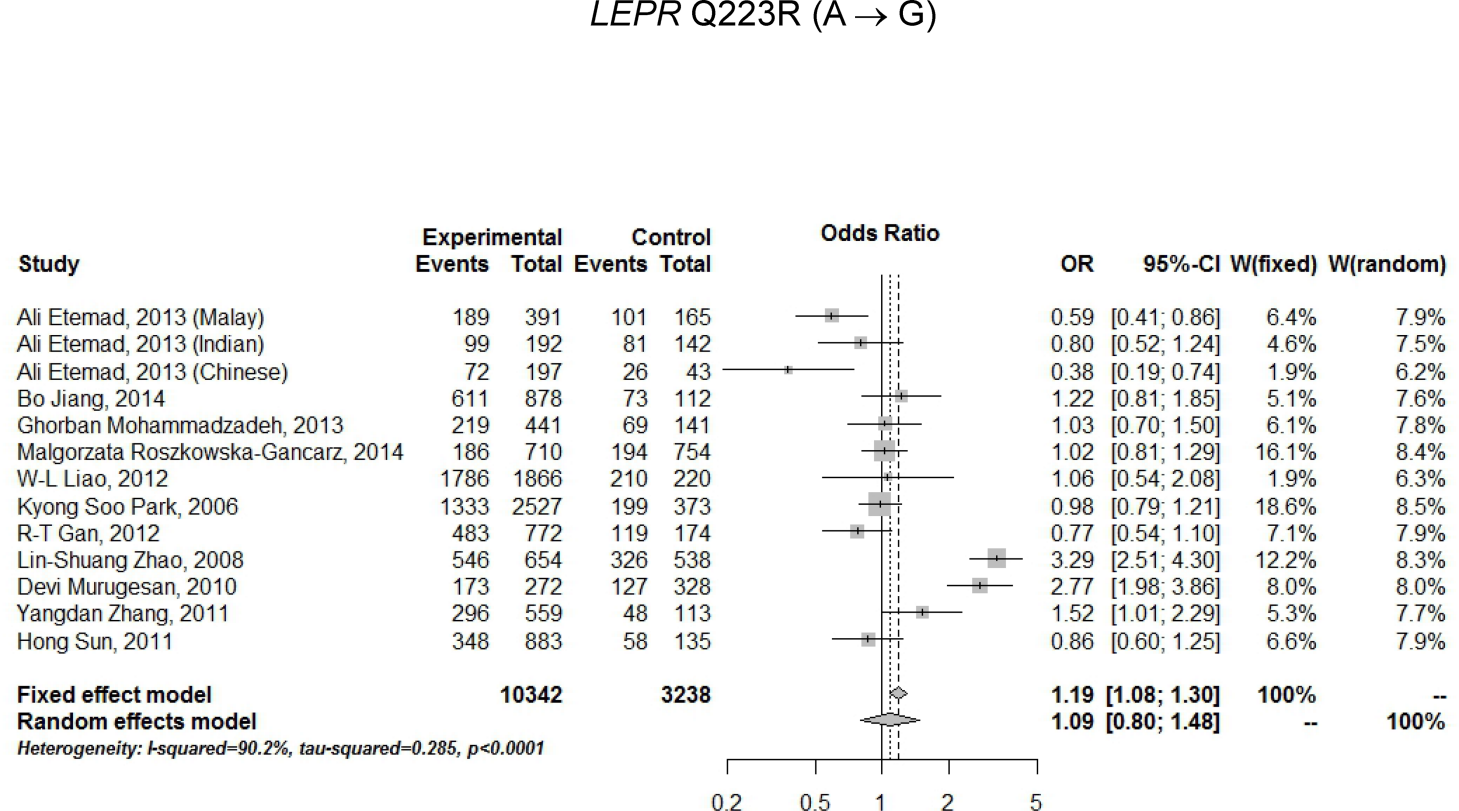
Forest plot for association of *LEPR* Q223R polymorphism with T2D risk under an allele model in total sample (n = 13 studies, random effects model).

**Fig 4:**
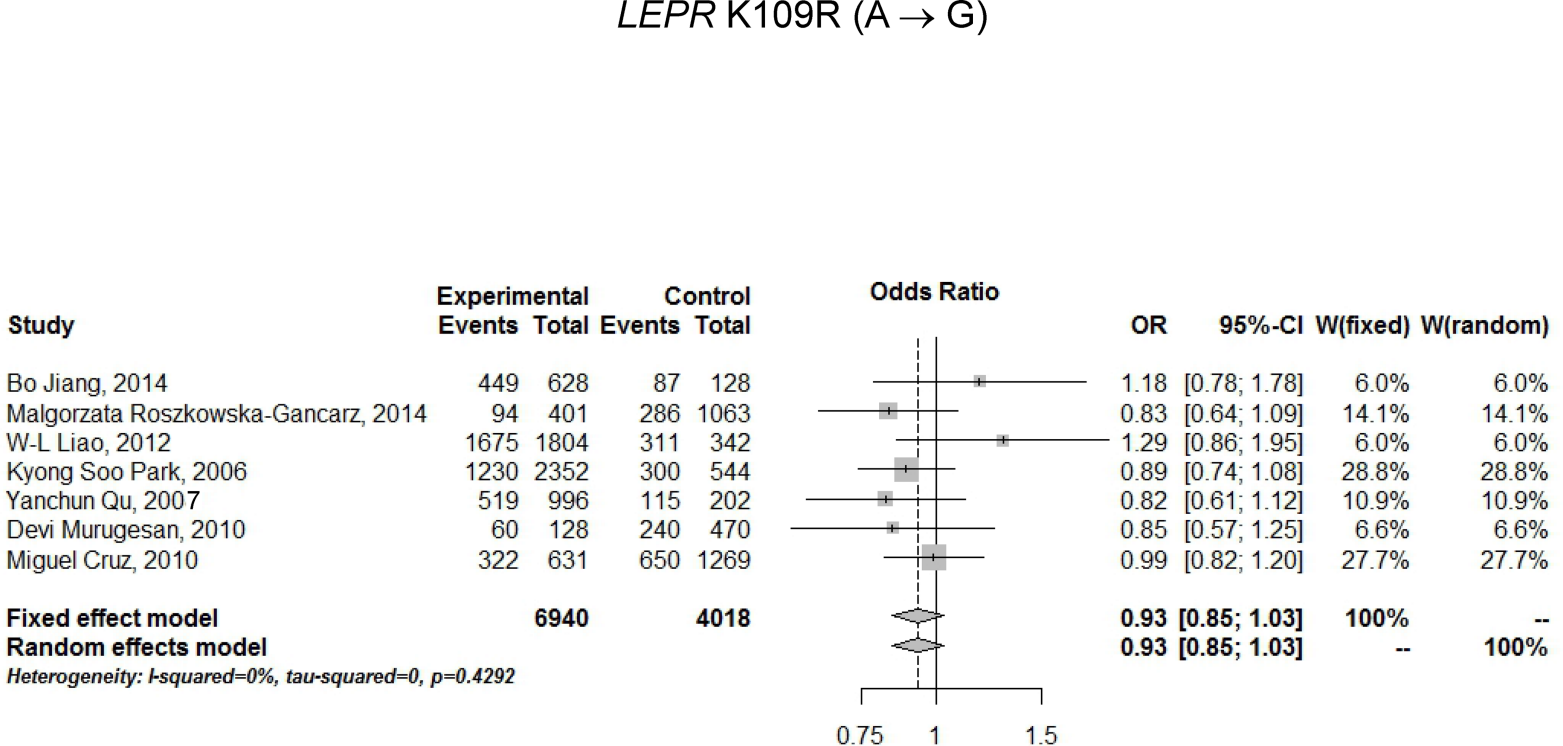
Forest plot for association of *LEPR* K109R polymorphism with T2D risk under an allele model in total sample (n = 7 studies, fixed effects model).

For assessing the relationship between *LEPR* Q223R polymorphism and T2D risk, a total of 13 studies (11 articles) were included (Table 3) and a random effects model was employed because of the presence of significant heterogeneity. Under an allelic model, a comparison of G vs. A produced an OR of 1.09 (95% CI: 0.80–1.48), which was not statistically significant (P-value = 0.5989) (Table 3 and Fig 3). Under genotypic models, comparisons of GG vs. AA, AG vs. AA, GG/AG vs. AA, and GG vs. AG/AA gave rise to ORs of 1.20, 1.08, 1.13, and 1.13 with P-values of 0.5741, 0.8177, 0.6871, and 0.3650, respectively, which also did not attain statistical significance. For assessing the relationship between *LEPR* K109R polymorphism and T2D risk, a total of 7 studies (7 articles) were included (Table 4) and a fixed effects model was employed because of a lack of significant heterogeneity. Under an allelic model, a comparison of G vs. A produced an OR of 0.93 (95% CI: 0.85–1.03), which did not reach statistical significance (P-value = 0.1868) (Table 4 and Fig 4). Under genotypic models, comparisons of GG vs. AA, AG vs. AA, GG/AG vs. AA, and GG vs. AG/AA produced ORs of 0.97 (95% CI: 0.74–1.26), 0.81 (95% CI: 0.67–0.97), 0.83 (95% CI: 0.70–0.99), and 0.99 (95% CI: 0.86–1.17) respectively, with P-values of 0.8087, 0.0207, 0.0384, and 0.8804 respectively, which all exceeded multiplicity-adjusted α = 0.05/5 = 0.01 with control for 5 genetic models.

**Table 3:** Meta-analysis results of the association between *LEPR* Q223R and T2D for 5 genetic models*.

Genetic model	# Studies	# Cases	# Controls	OR (95% CI)	P-value	I^2^	tau-squared	P_Heterogeneity_	Effects model
G vs. A	13	10342	3238	1.09 (0.80, 1.48)	0.5989	90.20%	0.285	< 0.0001	Random
GG vs. AA	13	4258	706	1.20 (0.64, 2.26)	0.5741	86.10%	1.032	< 0.0001	Random
AG vs. AA	13	1826	706	1.08 (0.58, 2.02)	0.8177	82.90%	0.9165	< 0.0001	Random
GG/AG vs. AA	13	6084	706	1.13 (0.61, 2.10)	0.6871	88.00%	0.9783	< 0.0001	Random
GG vs. AG/AA	13	4258	2532	1.13 (0.87, 1.45)	0.365	75.40%	0.1573	< 0.0001	Random

*LEPR Q223R is an A→G mutation (i.e., CAG→CGG) in exon 6, such that A is the wild-type allele, and G is the mutant allele.

Abbreviations: CI, confidence interval; OR, odds ratio.

**Table 4:** Meta-analysis results of the association between *LEPR* K109R and T2D for 5 genetic models*.

Genetic model	# Studies	# Cases	# Controls	OR (95% CI)	P-value	I^2^	tau-squared	P_Heterogeneity_	Effects model
G vs. A	7	6940	4018	0.93 (0.85, 1.03)	0.1868	0.00%	0	0.4292	Fixed
GG vs. AA	7	2563	1102	0.97 (0.74, 1.26)	0.8087	0.00%	0	0.8206	Fixed
AG vs. AA	7	1814	1102	0.81 (0.67, 0.97)	0.0207	0.00%	0	0.7008	Fixed
GG/AG vs. AA	7	4377	1102	0.83 (0.70, 0.99)	0.0384	0.00%	0	0.7389	Fixed
GG vs. AG/AA	7	2563	2916	0.99 (0.86, 1.17)	0.8804	8.50%	0.0041	0.3635	Fixed

*LEPR K109R is an A→G mutation (i.e., AAG→AGG) in exon 4, such that A is the wild-type allele, and G is the mutant allele.

Abbreviations: CI, confidence interval; OR, odds ratio.

### Test of heterogeneity

In the pooled analysis, for *LEPR* Q223R, a significant heterogeneity was detected for comparisons under 5 different genetic models, i.e., G vs. A, GG vs. AA, AG vs. AA, GG/AG vs. AA, and GG vs. AG/AA, such that I^2^ was 90.20%, 86.10%, 82.90%, 88.00%, and 75.40%, respectively (P-value for heterogeneity < multiplicity-corrected α = 0.05/5 = 0.01 for considering 5 genetic models), as shown in Table 3. Therefore, a random effects model was chosen to estimate this SNP’s pooled OR. For *LEPR* K109R, no statistically significant heterogeneity was detected for comparisons under 5 different genetic models, i.e., G vs. A, GG vs. AA, AG vs. AA, GG/AG vs. AA, and GG vs. AG/AA, such that I^2^s ranged from 0.00% to 13.60%, and P-values for heterogeneity ranged from 0.3274 to 0.8044, which exceeded multiplicity-corrected α = 0.05/5 = 0.01, as shown in Table 4. Because I^2^ was under 50% and P-values for heterogeneity were not significant for all these genetic models, a fixed effects model was applied in estimating this SNP’s pooled effect.

### Subgroup analysis

To explore sources of heterogeneity across studies, subgroup analyses by ethnicity (i.e., Chinese populations vs. non-Chinese populations) were conducted. For *LEPR* Q223R, 7 studies were performed in Chinese populations. Under genotypic models, a significant heterogeneity was detected for comparisons under 5 different genetic models, i.e., G vs. A, GG vs. AA, AG vs. AA, GG/AG vs. AA, and GG vs. AG/AA, such that I^2^ was 91.70%, 86.00%, 82.10%, 87.70%, and 78.80%, respectively (P-value for heterogeneity < multiplicity-adjusted α = 0.01 for each comparison), as shown in Table 5. For this SNP (i.e., Q223R), 6 studies were performed in non-Chinese populations. Under 5 different genetic models, i.e., G vs. A, GG vs. AA, AG vs. AA, GG/AG vs. AA, and GG vs. AG/AA, respectively (I^2^ was 88.50%, 85.10%, 78.50%, 84.90%, and 73.50%, and P-value for heterogeneity < multiplicity-adjusted α = 0.01 for each comparison), as shown in Table 6. Therefore, a random effects model was employed under each of these 5 genetic models in Chinese and non-Chinese populations, respectively. Pooled ORs (95% CIs) in Chinese populations had a range from 1.09 (95% CI: 0.31–3.88) to 1.17 (95% CI: 0.35–3.89) with P-values ranged 0.5476–0.8944 (Table 5; and the pooled effect under an allele model were displayed in a forest plot shown in S3 Fig) and in non-Chinese populations had a range from 0.98 (95% CI: 0.51–1.86) to 1.20 (95% CI: 0.58–2.47) with P-values ranged 0.5816–0.9436 (Table 6; and the pooled effect under an allele model were displayed in a forest plot shown in S4 Fig). For *LEPR* K109R, three studies were performed in Chinese populations (Table 7). Under each of 5 genetic models, no significant heterogeneity was detected (I^2^ ranged from 0% to 55.10%, and P-value for heterogeneity ranged from 0.1078 to 0.4121). For this SNP, 4 studies were performed in non-Chinese populations (Table 8). Under each of 5 genetic models, no significant heterogeneity was detected (I^2^ was consistently 0.00% for each comparison, and P-value for heterogeneity ranged from 0.5877 to 0.7808). Therefore, a fixed effects model was employed under each of these 5 genetic models in Chinese and non-Chinese populations, respectively. Pooled ORs (95% CIs) in Chinese populations had a range from 0.96 (95% CI: 0.45–2.03) to 1.03 (95% CI: 0.81–1.31) with P-values ranged 0.8044–0.959 (Table 7; and the pooled effect under an allele model were displayed in a forest plot shown in S5 Fig) and in non-Chinese populations had a range from 0.80 (95% CI: 0.66–0.96) to 0.97 (95% CI: 0.73–1.29) with P-values ranged 0.0167–0.8284 (Table 8; and the pooled effect under an allele model were displayed in a forest plot shown in S6 Fig).

**Table 5:** Meta-analysis results of the association between *LEPR* Q223R and T2D for 5 genetic models in Chinese population*.

Genetic model	# Studies	# Cases	# Controls	OR (95% CI)	P-value	I^2^	tau-squared	P_Heterogeneity_	Effects model
G vs. A	7	5809	1335	1.10 (0.65, 1.88)	0.722	91.70%	0.4645	< 0.0001	Random
GG vs. AA	7	2487	250	1.17 (0.35, 3.89)	0.7927	86.00%	2.067	< 0.0001	Random
AG vs. AA	7	835	250	1.09 (0.31, 3.88)	0.8944	82.10%	2.144	< 0.0001	Random
GG/AG vs. AA	7	3322	250	1.15 (0.33, 4.00)	0.8264	87.70%	2.28	< 0.0001	Random
GG vs. AG/AA	7	2487	1085	1.13 (0.75, 1.71)	0.5476	78.80%	0.2298	< 0.0001	Random

*LEPR Q223R is an A→G mutation (i.e., CAG→CGG) in exon 6, such that A is the wild-type allele, and G is the mutant allele.

Abbreviations: CI, confidence interval; OR, odds ratio.

**Table 6:** Meta-analysis results of the association between *LEPR* Q223R and T2D for 5 genetic models in Non-Chinese population*.

Genetic model	# Studies	# Cases	# Controls	OR (95% CI)	P-value	I^2^	tau-squared	P_Heterogeneity_	Effects model
G vs. A	6	4533	1903	1.06 (0.73, 1.54)	0.7679	88.50%	0.1887	< 0.0001	Random
GG vs. AA	6	1771	456	1.20 (0.58, 2.47)	0.6257	85.10%	0.6731	< 0.0001	Random
AG vs. AA	6	991	456	0.98 (0.51, 1.86)	0.9436	78.50%	0.4766	0.0003	Random
GG/AG vs. AA	6	2762	456	1.10 (0.57, 2.10)	0.7803	84.90%	0.5272	< 0.0001	Random
GG vs. AG/AA	6	1771	1447	1.10 (0.78, 1.55)	0.5816	73.50%	0.1283	0.002	Random

*LEPR Q223R is an A→G mutation (i.e., CAG→CGG) in exon 6, such that A is the wild-type allele, and G is the mutant allele.

Abbreviations: CI, confidence interval; OR, odds ratio.

**Table 7:** Meta-analysis results of the association between *LEPR* K109R and T2D for 5 genetic models in Chinese population*.

Genetic model	# Studies	# Cases	# Controls	OR (95% CI)	P-value	I^2^	tau-squared	P_Heterogeneity_	Effects model
G vs. A	3	3428	672	1.02 (0.83, 1.26)	0.8574	45.80%	0.0305	0.1579	Fixed
GG vs. AA	3	1427	49	0.96 (0.45, 2.03)	0.9115	0.00%	0	0.4025	Fixed
AG vs. AA	3	574	49	1.02 (0.47, 2.20)	0.959	0.00%	0	0.4086	Fixed
GG/AG vs. AA	3	2001	49	0.97 (0.46, 2.05)	0.9414	0.00%	0	0.4121	Fixed
GG vs. AG/AA	3	1427	623	1.03 (0.81, 1.31)	0.8044	55.10%	0.0586	0.1078	Fixed

*LEPR K109R is an A→G mutation (i.e., AAG→AGG) in exon 4, such that A is the wild-type allele, and G is the mutant allele.

Abbreviations: CI, confidence interval; OR, odds ratio.

**Table 8:** Meta-analysis results of the association between *LEPR* K109R and T2D for 5 genetic models in Non-Chinese population*.

Genetic model	# Studies	# Cases	# Controls	OR (95% CI)	P-value	I^2^	tau-squared	P_Heterogeneity_	Effects model
G vs. A	4	3512	3346	0.91 (0.81, 1.02)	0.1094	0.00%	0	0.7049	Fixed
GG vs. AA	4	1136	1053	0.97 (0.73, 1.29)	0.8284	0.00%	0	0.7808	Fixed
AG vs. AA	4	1240	1053	0.80 (0.66, 0.96)	0.0167	0.00%	0	0.6474	Fixed
GG/AG vs. AA	4	2376	1053	0.83 (0.69, 0.99)	0.0348	0.00%	0	0.6608	Fixed
GG vs. AG/AA	4	1136	2293	0.97 (0.81, 1.16)	0.7098	0.00%	0	0.5877	Fixed

*LEPR K109R is an A→G mutation (i.e., AAG→AGG) in exon 4, such that A is the wild-type allele, and G is the mutant allele.

Abbreviations: CI, confidence interval; OR, odds ratio.

### Sensitivity analysis

In order to assess the influence of each individual study on the pooled OR, we performed a sensitivity analysis by excluding each single study involved in the meta-analysis one at a time. For *LEPR* Q223R, the pooled ORs (95% CIs) ranged from 0.99 (95% CI: 0.78–1.27) to 1.17 (95% CI: 0.86–1.59) under an allelic model (Table 9), which was not dramatically changed from a pooled OR of 1.09 (95% CI: 0.80–1.48) under the same genetic model in the total sample (Table 3). For *LEPR* K109R, the pooled ORs (95% CIs) ranged from 0.91 (95% CI: 0.81–1.03) to 0.95 (95% CI: 0.85–1.06) under an allelic model (Table 10), which was not substantially altered from a pooled OR of 0.93 (95% CI: 0.85–1.03) under the same genetic model in the total sample (Table 4). These findings show that our results were statistically robust for both of these two polymorphisms.

**Table 9:** Sensitivity analysis results of the association between *LEPR* Q223R and T2D for allelic model*.

Study omitted	# Studies	OR (95% CI)	P-value	I^2^	P_Heterogeneity_	Effects model
Etemad, 2013 (Malays)	12	1.15 (0.83, 1.57)	0.4000	89.90%	<0.0001	Random
Etemad, 2013 (Chinese)	12	1.17 (0.86, 1.59)	0.3307	90.10%	<0.0001	Random
Etemad, 2013 (India)	12	1.11 (0.80, 1.55)	0.5210	90.80%	<0.0001	Random
Jiang, 2014	12	1.08 (0.77, 1.50)	0.6708	91.00%	<0.0001	Random
Mohammadzadeh, 2013	12	1.09 (0.78, 1.53)	0.6125	91.00%	<0.0001	Random
Roszkowska-Gancarz, 2014	12	1.09 (0.77, 1.55)	0.6329	90.90%	<0.0001	Random
Liao, 2012	12	1.09 (0.79, 1.51)	0.6117	91.00%	<0.0001	Random
Park, 2006	12	1.09 (0.77, 1.56)	0.6165	90.80%	<0.0001	Random
Gan, 2012	12	1.12 (0.80, 1.56)	0.5049	90.60%	<0.0001	Random
Zhao, 2008a	12	0.99 (0.78, 1.27)	0.9542	81.70%	<0.0001	Random
Murugesan, 2010	12	1.00 (0.74, 1.36)	0.9748	88.50%	<0.0001	Random
Zhang, 2011	12	1.06 (0.76, 1.47)	0.7488	90.90%	<0.0001	Random
Sun, 2011	12	1.11 (0.79, 1.54)	0.5481	90.80%	<0.0001	Random

*LEPR Q223R is an A→G mutation (i.e., CAG→CGG) in exon 6, such that A is the wild-type allele, and G is the mutant allele.

Abbreviations: CI, confidence interval; OR, odds ratio.

**Table 10:** Sensitivity analysis results of the association between *LEPR* K109R and T2D for allelic model*.

Study omitted	# Studies	OR (95% CI)	P-value	I^2^	P_Heterogeneity_	Effects model
Jiang, 2014	6	0.92 (0.83, 1.02)	0.1178	0.00%	0.4663	Fixed
Roszkowska-Gancarz, 2014	6	0.95 (0.85, 1.06)	0.3794	2.00%	0.4035	Fixed
Liao, 2012	6	0.92 (0.83, 1.02)	0.0943	0.00%	0.6427	Fixed
Park, 2006	6	0.95 (0.85, 1.07)	0.4229	10.80%	0.3463	Fixed
Qu, 2007	6	0.95 (0.85, 1.06)	0.3362	3.90%	0.3919	Fixed
Murugesan, 2010	6	0.94 (0.85, 1.04)	0.2531	11.90%	0.3388	Fixed
Cruz, 2010	6	0.91 (0.81, 1.03)	0.1326	7.80%	0.3666	Fixed

*LEPR K109R is an A→G mutation (i.e., AAG→AGG) in exon 4, such that A is the wild-type allele, and G is the mutant allele.

Abbreviations: CI, confidence interval; OR, odds ratio.

### Publication bias evaluation

**Fig 5:**
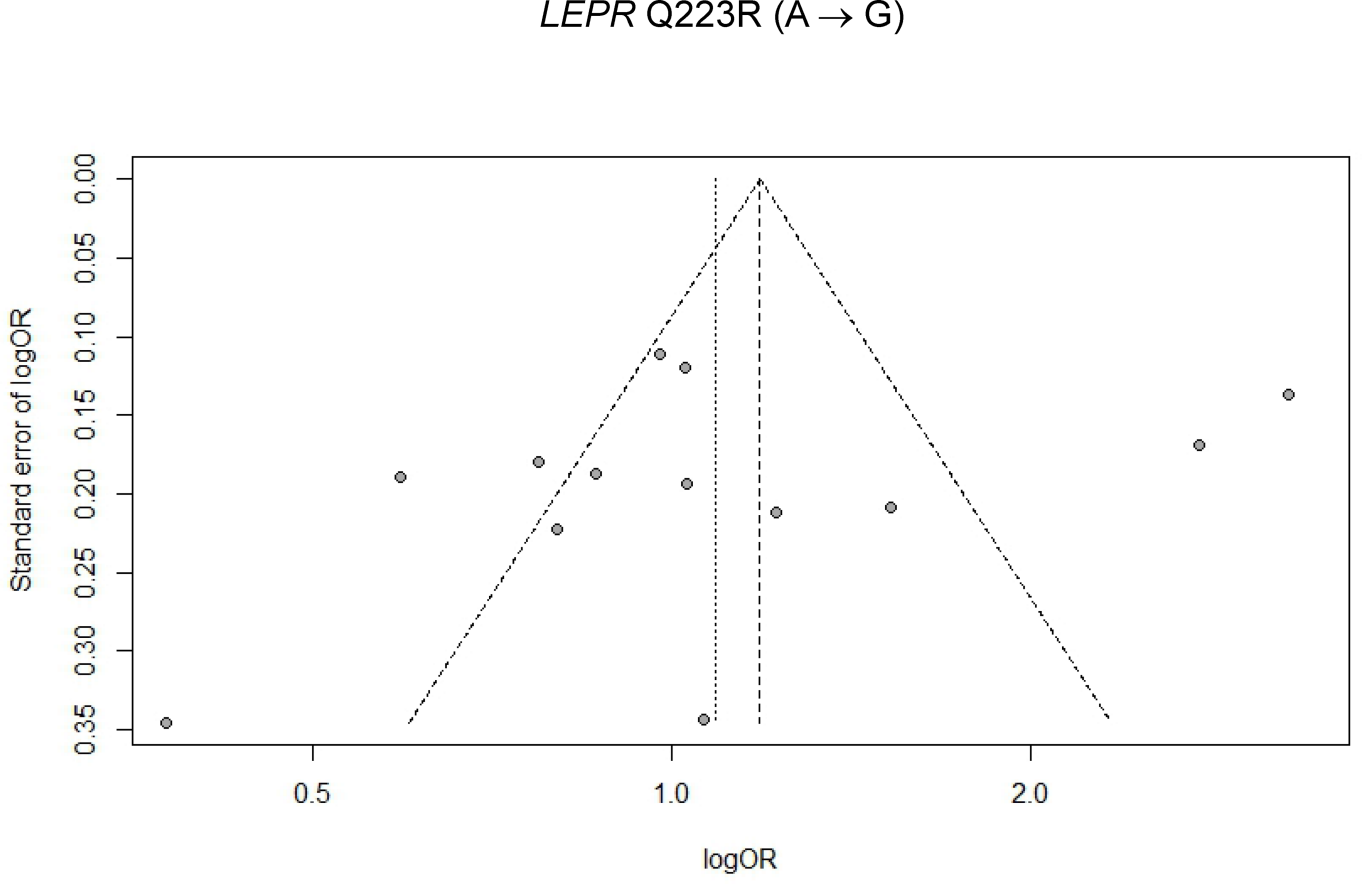
Funnel plot for association of *LEPR* Q223R polymorphism with T2D risk under an allele model in total sample (n = 13 studies).

**Fig 6:**
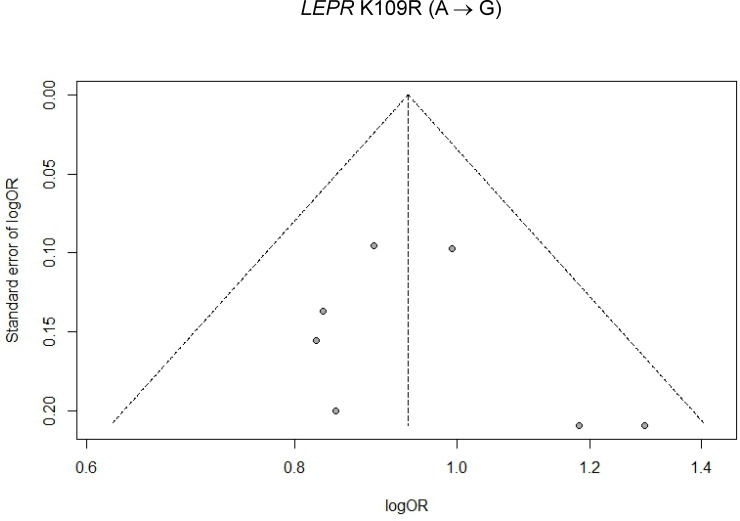
Funnel plot for association of *LEPR* K109R polymorphism with T2D risk under an allele model in total sample (n = 7 studies).

Visual inspections of respective funnel plots revealed no obvious asymmetry for associations of *LEPR* Q223R and T2D and *LEPR* K109R and T2D in total sample (Figs 5 and 6), Chinese populations (S7 and S8 Figs), and non-Chinese populations (S9 and S10 Figs), respectively. Begg and Mazumdar adjusted rank correlation test and Egger’s linear regression test were used to assess the publication bias for each SNP. No significant publication bias was observed in this meta-analysis [For *LEPR* Q223R: (1) an allele model (G vs. A): Begg and Mazumdar’s P-value = 0.7650, Egger’s P-value = 0.1932; (2) a homozygote model (GG vs. AA): Begg and Mazumdar’s P-value = 0.3674, Egger’s P-value = 0.5606; (3) a heterozygote model (AG vs. AA): Begg and Mazumdar’s P-value = 1.0000, Egger’s P-value = 0.2857; (4) a dominant model (GG+AG vs. AA): Begg and Mazumdar’s P-value = 0.5098, Egger’s P-value = 0.5570; and (5) a recessive model (GG vs. AG+AA): Begg and Mazumdar’s P-value = 0.9524, Egger’s P-value = 0.4236. For *LEPR* K109R: (1) an allele model (G vs. A): Begg and Mazumdar’s P-value = 0.2389, Egger’s P-value = 0.0463; (2) a homozygote model (GG vs. AA): Begg and Mazumdar’s P-value = 0.5619, Egger’s P-value = 0.8058; (3) a heterozygote model (AG vs. AA): Begg and Mazumdar’s P-value = 0.7726, Egger’s P-value = 0.8902; (4) a dominant model (GG+AG vs. AA): Begg and Mazumdar’s P-value = 0.7726, Egger’s P-value = 0.6220; and (5) a recessive model (GG vs. AG+AA): Begg and Mazumdar’s P-value = 0.7726, Egger’s P-value = 0.9867]. All the above P-values exceeded multiplicity-adjusted α = 0.05/5 = 0.01.

### Bioinformatics analysis

Based on 7 different *in silico* tools, both *LEPR* Q223Rand K109R are predicted to exert a low impact on protein function (by Mutation Assessor), to be evolutionarily more acceptable (by BLOSUM62) neutral (by PROVEAN, PANTHER, SNPs&GO, and SNPs3D) and benign (by PolyPhen-2) (Table 11).

**Table 11:** *In silico* predicted functional effects of *LEPR* Q223R and K109R*.

Gene Symbol	SNP ID (WT/MUT alleles; AA change)	Mutation Assessor FI score (Prediction)	BLOSUM62 score (Prediction)	PROVEAN delta Score (Prediction)	PolyPhen-2 score (Prediction)	PANTHER subPSEC score [P_deleterious_ (Prediction)]	SNPs&GO Disease probability [RI] Score (Prediction)	SNPs3D SVM score (Prediction)
*LEPR*	Q223R (A/G; rs1137101)	1.32 (low impact)	1.00 (evolutionarily more acceptable)	-1.271 (neutral)	0.282 (benign)	-1.8785 [0.24573 (neutral)]	0.110 [8] (neutral)	3.19 (neutral)
*LEPR*	K109R (A/G; rs1137100)	1.67 (low impact)	2.00 (evolutionarily more acceptable)	-0.378 (neutral)	0.077 (benign)	-1.75027 [0.22275 (neutral)]	0.038 [9] (neutral)	1.79 (neutral)

*LEPR Q223R is an A→G mutation (i.e., CAG→CGG) in exon 6, such that A is the wild-type allele, and G is the mutant allele.

*Abbreviations: AA, amino acid; BLOSUM, BLOcks SUbstitution Matrix; FI, functional impact; MUT, mutant; LEPR, leptin receptor; PANTHER, Protein ANalysis THrough Evolutionary Relationships; RI, reliability index; SNP, single nucleotide polymorphism; Polyphen-2; Polymorphism Phenotyping v2; PROVEAN, PROtein Variation Effect ANalyzer; subPSEC, subStitution Position-specific Evolutionary Conservation; SVM, support vector machine; WT, wild-type.

## Discussion

LEP, a pleiotropic hormone produced primarily by adipose tissue, plays an essential role in signaling energy status to the central nervous system (CNS), which has helped to redefine adipose tissue as an endocrine organ [60]. By binding to LEPRs expressed by neurons in CNS [61], leptin exerts its physiological effects on food intake, body weight, glucose and lipid metabolism, and regulation of immune function [15]. Although several independent studies identified significant associations between genetic variants of *LEPR* and obesity (e.g., [62, 63]), others did not (e.g., [58, 64]). Three meta-analysis studies (i.e., [65–67]) did not find significant relationships of *LEPR* polymorphisms with either obesity or obesity-related outcomes. In current study, 13 studies (11 articles; 4030 cases and 2844 controls) for Q223R, and 7 studies (7 articles; 3319 cases and 2465 controls) for K109R were included, which far exceed the sample size of any individual study. By employing 5 different genetic models to meta-analyze potential effects of these two missense SNPs on T2D risk, we did not detect statistically significant associations of either Q223R or K109R with T2D risk in either main analyses or subgroup analyses. Further, based on 7 software tools, both missense SNPs were predicted to be functionally neutral and benign.

The VAFs for Chinese and non-Chinese populations for *LEPR* Q223R and K109R are not uniform across different ethnic groups. For Q223R, higher VAFs were observed in Chinese T2D cases (0.82) and controls (0.79) than in non-Chinese T2D cases (0.64) and controls (0.63), respectively (S1 Fig). Further, for K109R, higher VAFs were observed in Chinese T2D cases (0.83) and controls (0.82) than in non-Chinese T2D cases (0.40) and controls (0.42), respectively (S2 Fig). VAFs for both missense SNPs in Chinese populations of current study were similar to those reported in other studies, e,g., [61] and [68], which appear to be higher than in non-Chinese populations. As shown in Fan and Say (2014) [61], even among Asians, the respective allele frequencies of variant alleles R223 and R109 were notably higher in Chinese than Indians and Malays.

A comparison between the current meta-analysis and three other meta-analysis studies, i.e., Yang et al. (2016) [24], Liu et al. (2015) [69], Su et al. (2016) [70], is shown in Table 12. For Yang et al. (2016) [24], 7 *LEPR* gene’s molecular variants, i.e., Q223R (rs1137101), K109R (rs1137100), S343S (rs1805134, formerly rs3790419), N567N (rs2228301), K656N (rs1805094, formerly rs8179183), P1019P (rs1805096), and the 3’ UTR Ins/Del in T2D risk were assessed (11, 7, 1, 1, 5, 3, and 2 studies were included for them, respectively). However, only 5 *LEPR* polymorphisms, i.e., Q223R, K109R, K656N, P1019P and 3’ UTR Ins/Del, were meta-analyzed because only 1 article was found for each of S343S and N567N, respectively. For Liu et al. (2015) [69], only Q223R was studied, whereas for Su et al. (2016) [70], 4 *LEPR* polymorphisms, i.e., Q223R, K109R, K656N, and P1019P, were meta-analyzed. With respect to Q223R, our results were concordant with those of Liu et al. (2015) [69] and Su et al. (2016) [70] such that no statistically significant associations were found. However, significant association was found by Yang et al. (2016) [24]. With respect to K109R, our results were concordant with those of Yang et al. (2016) [24] and Su et al. (2016) [70], such that no significant relationship was found between this missense SNP and T2D risk. With respect to another *LEPR* missense SNP, i.e., K656N, which was meta-analyzed by Yang et al. (2016) [24] and Su et al. (2016) [70], 5 and 4 studies were included in each of these two meta-analysis studies, respectively, which limited their abilities to draw robust conclusions on them. Therefore, to ensure that there are sufficiently large numbers of individual studies (i.e., > 5) amenable to subgroup analyses, only Q223R and K109R were assessed in the current study. We found that neither of these two missense SNPs is significantly associated with T2D risk. Taken together, based on our careful assessments, for Yang et al. (2016) [24], Liu et al. (2015) [69], Su et al. (2016) [70], there are errors (i.e., the genotype count data were incorrectly assigned to at least one included study) in data extraction from individual studies (affecting all these three studies) (affecting all of [24], [69], and [69), and errors (i.e., included studies contain overlapping data) in the number of individual studies included for meta-analysis a SNP (affecting [24] and [69]) (Table 12).

**Table 12:** Comparison of methods and results of current study with three previously published meta-analysis studies*.

Category	Yang et al. (2016)	Liu et al. (2015)	Su et al. (2016)	Current study
**SNPs studied**	**Q223R**, **K109R**, **K656N**, **P1019P**, **3’ UTR**	**Q223R**	**Q223R**, **K109R**, **K656N**, **P1019P**	**Q223R**, **K109R**
**Databases searched**	PubMed, EMBASE	PubMed, EMBASE, Web of Science, and Chinese Biomedical Database (CBM)	PubMed, EMBASE, EBSCO, Web of Knowledge, CNKI, SinoMed, Chinese VIP Database, and Chinese Wanfang Database	PubMed, EMBASE, Cochrane Library, Google Scholar
**Genetic models applied for each SNP (Number of models)**	Allele, Homozygote, Dominant, Recessive (4)	Allele, Homozygote, Dominant, Recessive (4)	Allele, Homozygote, Dominant, Recessive (4)	Allele, Homozygote, Heterozygote, Dominant, Recessive (5)
**Number of studies (number of cases, number of controls) included for each SNP**	**Q223R:** 11 studies (3,649 cases and 2,381 controls); **K109R:** 7 studies (3,536 cases and 2,268 controls); **K656N:** 5 studies (2,018 cases and 1,641 controls); **P1019P:** 3 studies (753 cases and 767 controls); **3’ UTR:** 2 studies (544 cases and 690 controls)	**Q223R:** 16 studies (4,471 cases and 3,356 controls)	**Q223R:** 18 studies (15,495 cases and 12,018 controls); **K109R:** 6 studies (8,049 cases and 5,943 controls); **K656N:** 4 studies (4,266 cases and 4,971 controls); **P1019P:** 6 studies (3,450 cases and 2,628 controls)	**Q223R:** 13 studies (4,030 cases and 2,844 controls); **K109R:** 7 studies (3,319 cases and 2,465 controls)
**Data accrual**	Weaknesses in data extraction: (i) For **Q223R**, **K109R**, and **K656N**, the entire study sample of 752 women of Han et al. (2008) [85] is the women subsample of the Seoul National University Hospital (SNUH) data of Park et al. (2006) [53] (containing both men and women subsamples), and therefore, the study of Han et al. (2008) [85] should be removed because these two individual studies are overlapping. (ii) The allele codings for both **Q223R** and **K109R** of Murugesan et al. (2010) [55] are incorrect: the Q223 (A allele) and R223 (G allele) codings for Murugesan et al. (2010) [55] shall be switched (e.g., in Tables 1 and 2 and Figs 2 and 3 of Yang et al. (2016) [24]). (iii) For **P1019P**, the SNP name shall be corrected to rs1805096, rather than rs62589000 [According to NCBI dbSNP (https://www.ncbi.nlm.nih.gov/snp), rs62589000 is a chromosome X SNP]. (iv) For **Q223R**, the study of Takahashi-Yasuno (2004) [86] was included just for the Dominant model, but not the other 3 genetic models, because no genotype count data for the three genotypes (i.e., AA, AG, and GG), were available to the authors of Yang et al. (2016) [24]. (v) For **K109R**, the study of Cruz et al. (2010) [59] lacked genotype data for count data for the three genotypes (i.e., AA, AG, and GG).	Weaknesses in data extraction: for **Q223R**, the study sample of Zhao et al. (2008b) [87] [i.e., Reference [7] of the study, i.e., Liu et al. (2015)] and the study sample of Zhao et al. (2008a) [54] [i.e., Reference [6] of the study, i.e., Liu et al. (2015)] are the same study, and therefore, the study of Zhao et al. (2008b) [87] [i.e., Reference [7] of the study, i.e., Liu et al. (2015)] should be removed [e.g., in Tables 1 and 2, and Fig 1 of the study, i.e., Liu et al. (2015)].	Weaknesses in data extraction: the allele codings for both **Q223R** and **K109R** of Murugesan et al. (2010) [55] are incorrect: the Q223 (A allele) and R223 (G allele) codings used for Murugesan et al. (2010) [55] shall be switched [e.g., in Tables 2 and 4, and S10-S13 Figs of the study, i.e., Su et al., (2016)].	All data extraction problems of three previously published meta-analysis studies were addressed.
**Results for each SNP**	[The results shown were those reported by the study, which included incorrectly extracted data as indicated in above “**Data accrual**” section]: **Q223R** P-values (Table 2 of the study): Allele: **< 0.0001**, Homozygote: **< 0.0001**, Dominant: **0.007**, Recessive: **< 0.0001**; **K109R** P-values (Table 2 of the study): Allele: 0.73, Homozygote: 0.44, Dominant: 0.86, Recessive: 0.39; **K656N** P-values (Table 2 of the study): Allele: 0.38, Homozygote: 0.53, Dominant: 0.36, Recessive: 0.5; **P1019P** P-values (Table 2 of the study): Allele: 0.13, Homozygote: **0.01**, Dominant: 0.25, Recessive: **0.01**; **3’ UTR** P-value (Table 2 of the study): Allele: **0.001**, Homozygote: 0.84, Dominant: **0.008,** Recessive: 0.92.	[The results shown were those reported by the study, which included incorrectly extracted data as indicated in above “**Data accrual**” section]: **Q223R** P-value (Table 2 of the study): Allele: 0.457, Homozygote: 0.375, Dominant: 0.254, Recessive: 0.612	[The results shown were those reported by the study, which included incorrectly extracted data as indicated in above “**Data accrual**” section]: **Q223R** P-value (Table 4 of the study): Allele: 0.08, Homozygote: 0.30, Dominant: 0.20, Recessive: 0.19; **K109R** P-value (Table 4 of the study): Allele: 0.98, Homozygote: 0.55, Dominant: 0.39, Recessive: 0.75; **K656N** P-value (Table 4 of the study): Allele: 0.98, Homozygote: 0.89, Dominant: 0.86, Recessive: 0.94; **P1019P** P-value (Table 4 of the study): Allele: **0.0005**, Homozygote: **< 0.00001**, Dominant: **0.0002**, Recessive: **0.003**.	**Q223R** P-value (Table 2 of the study): Allele: 0.5989, Homozygote: 0.5741, Heterozygote: 0.8177, Dominant: 0.6871, Recessive: 0.365. **K109R** P-value (Table 3 of the study): Allele: 0.1868, Homozygote: 0.8087, Heterozygote: 0.0207, Dominant: 0.0384, Recessive: 0.8804.
**Subgroup analysis**	No	Yes	No	Yes
**Sensitivity analysis**	Yes	No	Yes	Yes
**Funnel plot**	Yes	Yes	No	Yes
**Begg and Mazumdar adjusted rank correlation test**	No	No	Yes	Yes
**Egger linear regression test**	Yes	Yes	Yes	Yes

*The multiplicity-corrected α for Yang et al. (2016) [24], Liu et al. (2015) [69], Su et al. (2016) [70] shall be adjusted according to the number of genetic models studied by each study, i.e., 0.05/4 = 0.0125, because each study has applied 4 different genetic models; the originally reported P-values were shown in bold font if the P-values were below this multiplicity-corrected α. The multiplicity-corrected α for the current study is adjusted according to the number of genetic models studied, i.e., 0.05/5 = 0.01, and if the P-value is below this multiplicity-corrected α, would be shown in bold font.

Caution should be taken when interpreting our results on the associations of gene polymorphisms with T2D. A significant heterogeneity was detected for Q223R (P-values for heterogeneity < multiplicity-corrected α = 0.05/5 = 0.01 for considering 5 genetic models (Table 3)], but not for K109R [range of P-values, 0.0205–0.6487, which were > multiplicity-corrected α = 0.05/5 = 0.01 (Table 4)] and subgroup analyses were conducted to explore reasons of heterogeneity. When stratified by ethnicity (i.e., Chinese vs non-Chinese populations), for Q223R, heterogeneity remained significant in each subgroup [P-values for heterogeneity < 0.0001 in Chinese populations (Table 5) and ≤ 0.002 non-Chinese populations (Table 6), respectively, which were all < multiplicity-corrected α = 0.05/5 = 0.01], and therefore, ethnicity did not appear to explain heterogeneity for Q223R. No heterogeneity was detected for K109R in either Chinese populations (Table 7) or non-Chinese populations (Table 8), because P-value for heterogeneity for each model was > multiplicity-corrected α = 0.05/5 = 0.01. In order to evaluate the influence of single studies on the overall estimate, a sensitivity analysis was performed by deleting each single study one at a time for allele model. The omission of any single study did not significantly alter pooled effect estimates for either Q223R (Table 9) or K109R (Table 10), suggesting that our meta-analysis results were both reliable and credible. For assessments of publication bias, funnel plots were generated and their symmetries were tested using Begg and Mazumdar rank correlation and Egger’s linear regression tests. Both tests revealed that no significant biases existed (P-values > 0.05 for all 5 genetic models for each SNP), and inspections of funnel plots also indicated no evidence of publication bias for the entire study sample [Fig 5 (Q223R) and Fig 6 (K109R)], and for either Chinese populations [S7 Fig (Q223R) and S8 Fig (K109R)] or non-Chinese populations [S9 Fig (Q223R) and S10 Fig (K109R)].

Our meta-analysis had several advantages: (1) Compared with the three previously published meta-analysis studies, i.e., Yang et al. (2016) [24], Liu et al. (2015) [69], Su et al. (2016) [70] their mistakes in data extraction were corrected and their weaknesses in considering 4 genetic models were well-addressed. (2) Both subgroup and sensitivity analyses were performed in the current study whereas only one of these two important types of analyses was employed by each of the three previously published meta-analysis studies (Table 12), which demonstrated that our results were statistically stable. (3) The current study applied both Begg and Mazumdar adjusted rank correlation test and Egger’s linear regression test whereas [of the three previously performed meta-analyses, only one study, i.e., Su et al. (2016) [70] employed both, but only for 4 genetic models], we did not detect any publication biases by funnel plot inspections in either main analyses or subgroup analyses, indicating that our results were unbiased. (4) In the current study, all included studies were of sufficiently high quality (i.e., NOS score ≥ 7), which all met our inclusion criteria. (5) To assess functional impacts of these two common missense SNPs, 7 *in silico* tools were applied, and their results were consistent with each other.

There are several limitations in the current study: (1) Our meta-analysis was based on unadjusted OR estimates due to a lack of individual participants’ data. There is an important potential source of type II error β in the inference that *LEPR* genetic variants does not contribute to diabetes-susceptibility in our meta-analysis. Some of the individual studies, e.g., Liao et al. (2012) [23] and Roszkowska-Gancarz et al. (2014) [52], which were included for meta-analysis of both Q223R and K109R, did not match body weight and age between cases and controls, or adjust computationally for these important covariates which are critical to penetrance of genes predisposing to T2D. Since T2DM is highly correlated with body weight and age, using thinner and younger control subjects compared to T2D cases (e.g., Etemad et al. (2013) [49]), could confound the estimate of a non-weight dependent T2DM effect of *LEPR* genetic variants. (2) The study examined two most widely studied missense SNPs of *LEPR* in T2D, i.e., Q223R (rs1137101) and K109R (rs1137100) which were in a moderate level of linkage disequilibrium (LD) (e.g., *r*^*2*^ = 0.3647 in Caucasians [71]), and haplotype-based association analysis could provide more statistical power than single SNP analysis [72–74]. (3) We applied a Bonferroni procedure to correct for the 5 genetic models tested, as in Wong et al. (2015) [75], and this procedure could be conservative. (4) The number of studies included in our meta-analysis, particularly the subgroup analyses according to ethnicity, was limited. (5) For Q223R, because individual studies had diverse population characteristics, significant between-study heterogeneity was observed, which could affect the precision of results, although the random effects model was applied in the presence of significant heterogeneity to pool ORs for this SNP. (6) T2D is polygenic and multifactorial, and there are a variety of possible genetic (> 80 genetic susceptibility loci have been identified [76], e.g., *TCF7L2*, *PPARG*), environmental (e.g., air pollution by nitrogen dioxide, PM_2.5_, and PM_10_ [77]), nutritional (e.g., dietary fiber, fat intake [78]), lifestyle (e.g., physical inactivity [79]) and sociodemographic (e.g., age, ethnicity, education [80]) risk factors involved in the etiology of this disease. Because the definition of T2D varies among the individual studies [The World Health Organization (WHO) and American Diabetes Association (ADA) represent the two most widely used criteria (Tables 1 and 2)], over- (i.e., too many) or under-(i.e., too few) inclusion of subjects could be a possibility for each study. (7) Potential gene-gene and gene-environment interactions may influence the associations of *LEPR* gene Q223R and K109R polymorphisms and T2D risk. (8) This meta-analysis focused only on articles published in the English and Chinese languages, and there may be other eligible studies that were published in other languages.

In conclusion, to the best of our knowledge, the current study is most up-to-date, robust, and unbiased, when compared to previously published meta-analysis studies (i.e., Yang et al. (2016) [24], Liu et al. (2015) [69], Su et al. (2016) [70]) in this field. Neither Q223R nor K109R was significantly associated with T2D risk in the current meta-analysis, and bioinformatics analysis predicted that both SNPs are functionally neutral and benign. Additional well-designed independent studies with sufficiently large sample sizes in various ethnicities could be conducted to confirm our findings.

## Supporting information

S1 File PRISMA checklist. PRISMA 2009 checklist.
(DOC)

S2 File Checklist. Meta-analysis of genetic association studies checklist.
(DOC)

S3 File Electronic search strategy and results. Electronic search strategy and results for PubMed, EMBASE, Cochrane Library, and Google Scholar.
(XLS)

S1 Fig Q223 allele frequencies of *LEPR* Q223R polymorphism for T2D cases and controls in Chinese (left panel) and non-Chinese (right panel) populations. (TIFF)

S2 Fig R109 allele frequencies of *LEPR* K109R polymorphism for T2D cases and controls in Chinese (left panel) and non-Chinese (right panel) populations. (TIFF)

S3 Fig Forest plot for association of *LEPR* Q223R polymorphism with T2D risk under an allele model in Chinese populations (n = 7 studies, random effects model). (TIFF)

S4 Fig Forest plot for association of *LEPR* Q223R polymorphism with T2D risk under an allele model in non-Chinese populations (n = 6 studies, random effects model). (TIFF)

S5 Fig Forest plot for association of *LEPR* K109R polymorphism with T2D risk under an allele model in Chinese populations (n = 3 studies, fixed effects model). (TIFF)

S6 Fig Forest plot for association of *LEPR* K109R polymorphism with T2D risk under an allele model in non-Chinese populations (n = 4 studies, fixed effects model). (TIFF)

S7 Fig Funnel plot for association of *LEPR* Q223R polymorphism with T2D risk under an allele model in Chinese populations (n = 7 studies). (TIFF)

S8 Fig Funnel plot for association of *LEPR* K109R polymorphism with T2D risk under an allele model in Chinese populations (n = 3 studies). (TIFF)

S9 Fig Funnel plot for association of *LEPR* Q223R polymorphism with T2D risk under an allele model in non-Chinese populations (n = 6 studies). (TIFF)

S10 Fig Funnel plot for association of *LEPR* K109R polymorphism with T2D risk under an allele model in non-Chinese populations (n = 4 studies). (TIFF)
