# Supplementary figures and images for: A meta-analysis of associations of LEPR Q223R and K109R polymorphisms with Type 2 diabetes risk
Source: PLoS One. 2018 Jan 2;13(1):e0189366. doi: 10.1371/journal.pone.0189366 (PMC5749718; doi:10.1371/journal.pone.0189366)

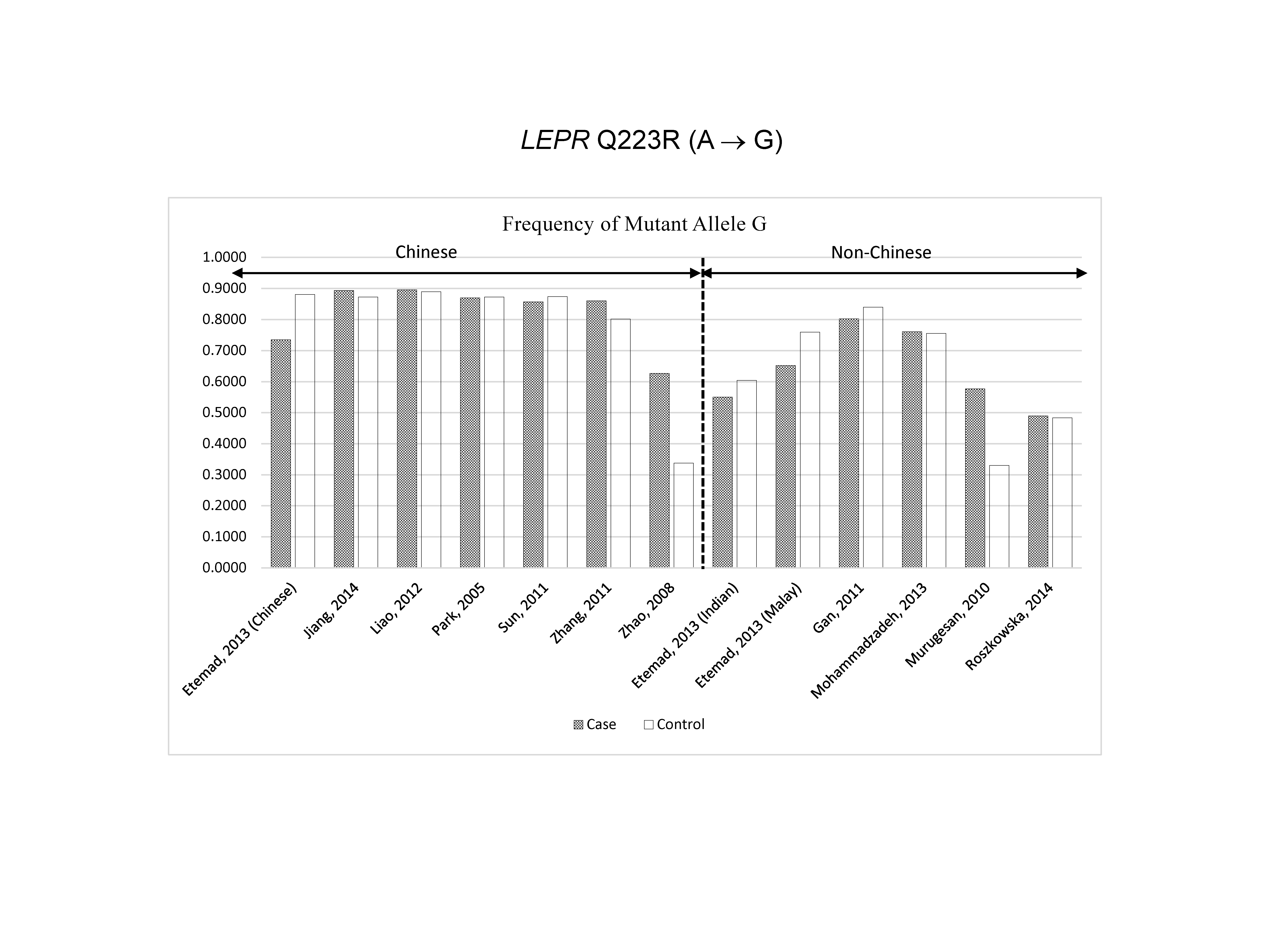

Supplement: S1 Fig — (TIFF) [file pone.0189366.s004.tiff]

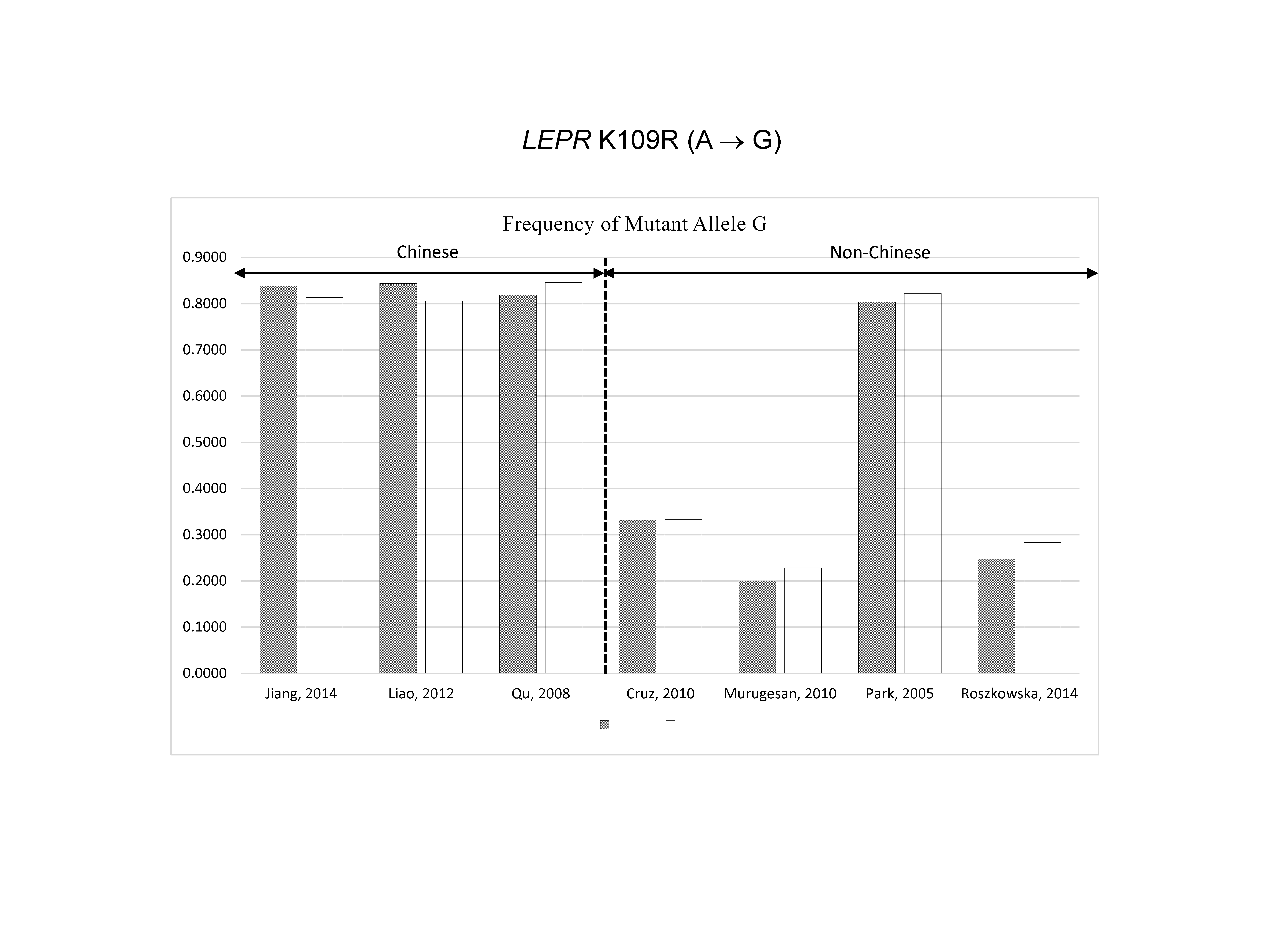

Supplement: S2 Fig — (TIFF) [file pone.0189366.s005.tiff]

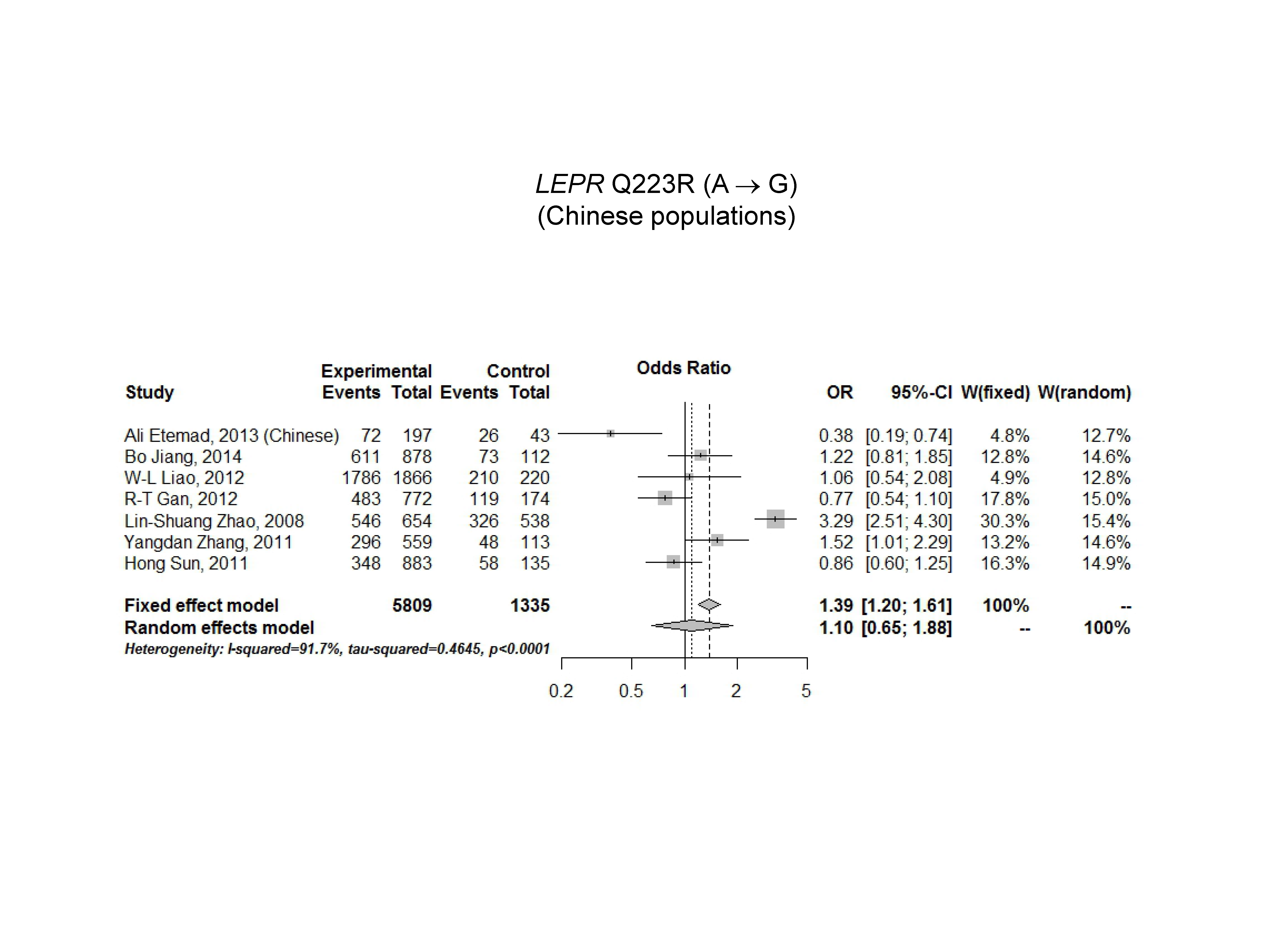

Supplement: S3 Fig — (TIFF) [file pone.0189366.s006.tiff]

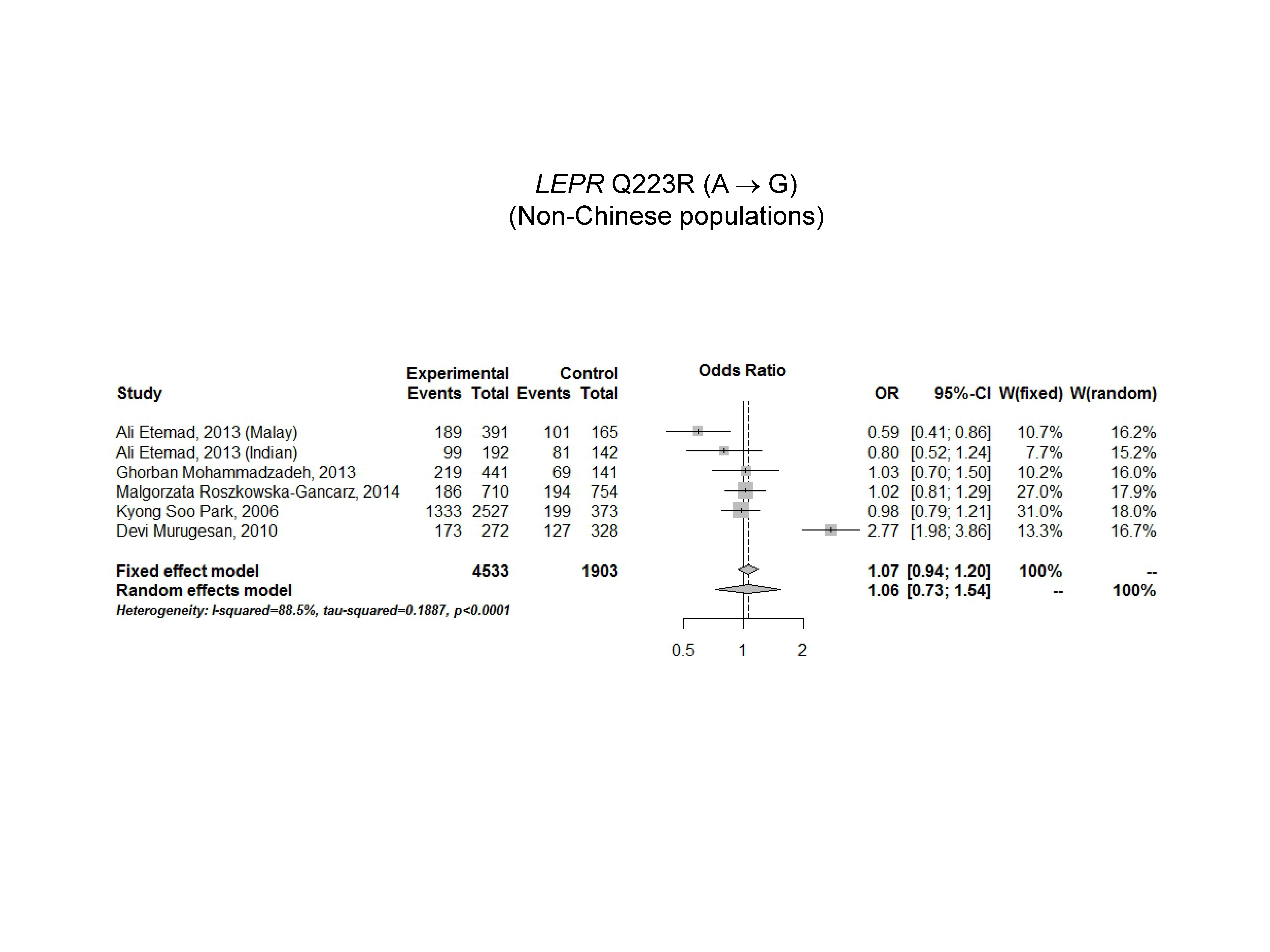

Supplement: S4 Fig — (TIFF) [file pone.0189366.s007.tiff]

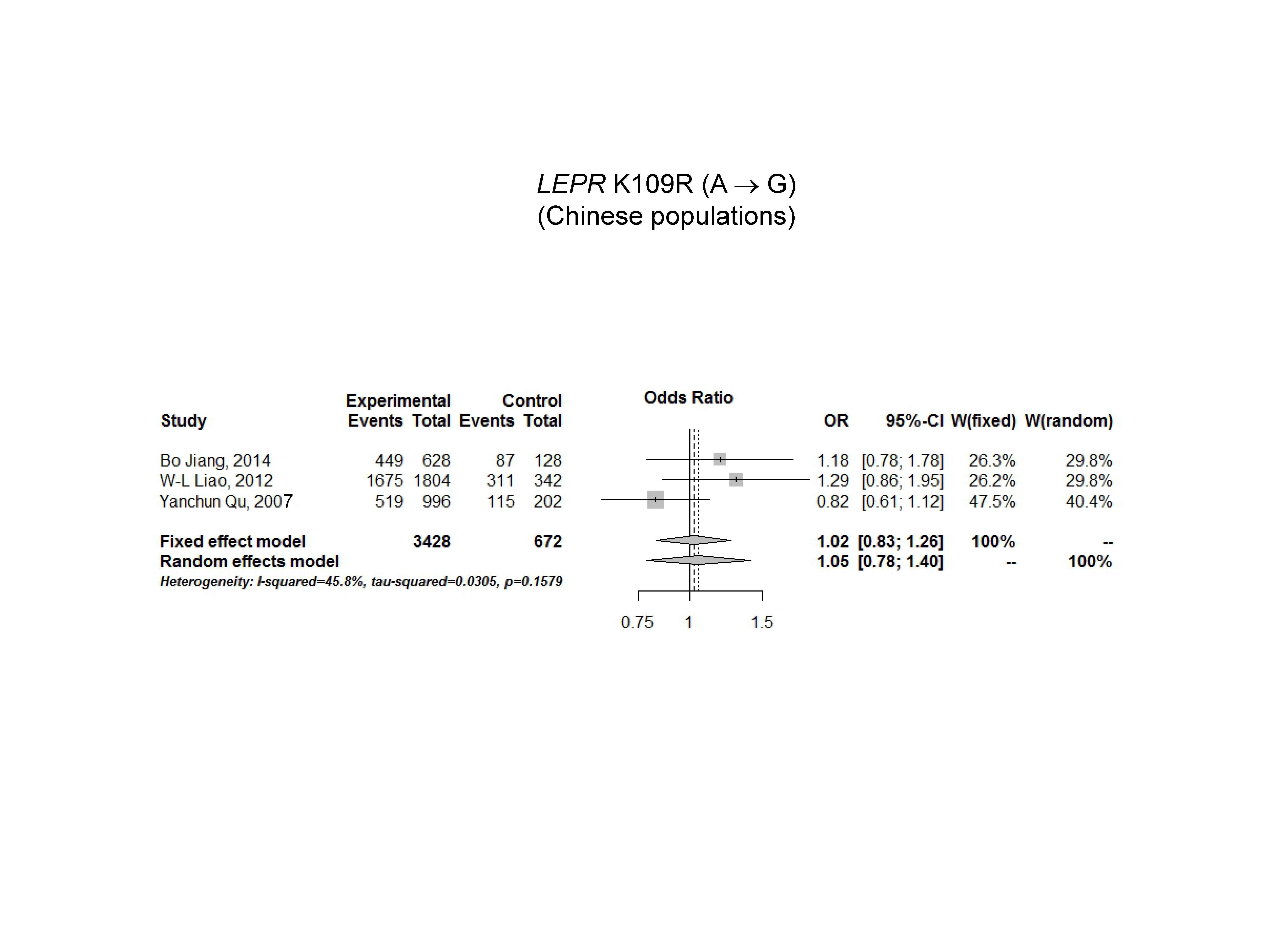

Supplement: S5 Fig — (TIFF) [file pone.0189366.s008.tiff]

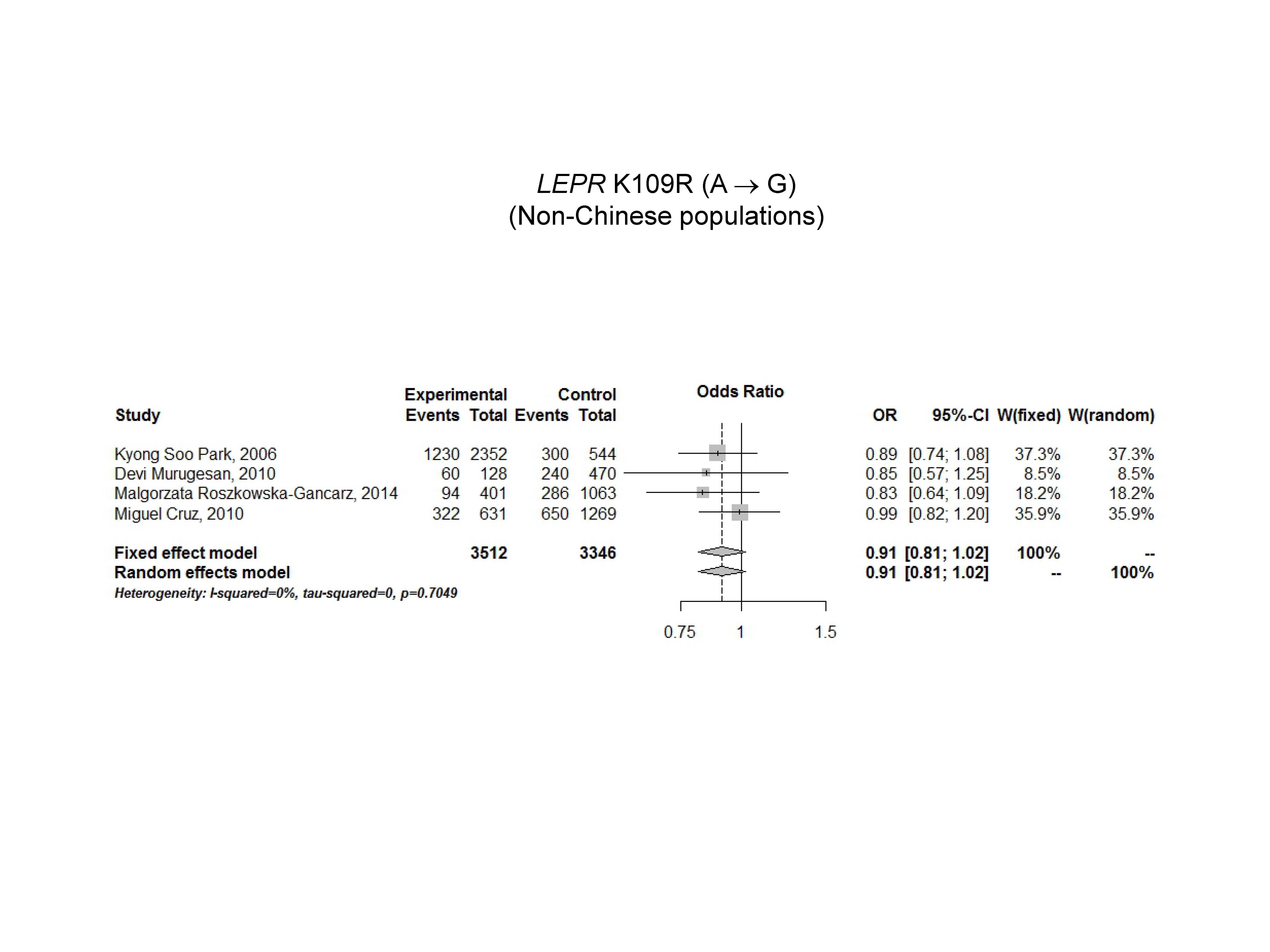

Supplement: S6 Fig — (TIFF) [file pone.0189366.s009.tiff]

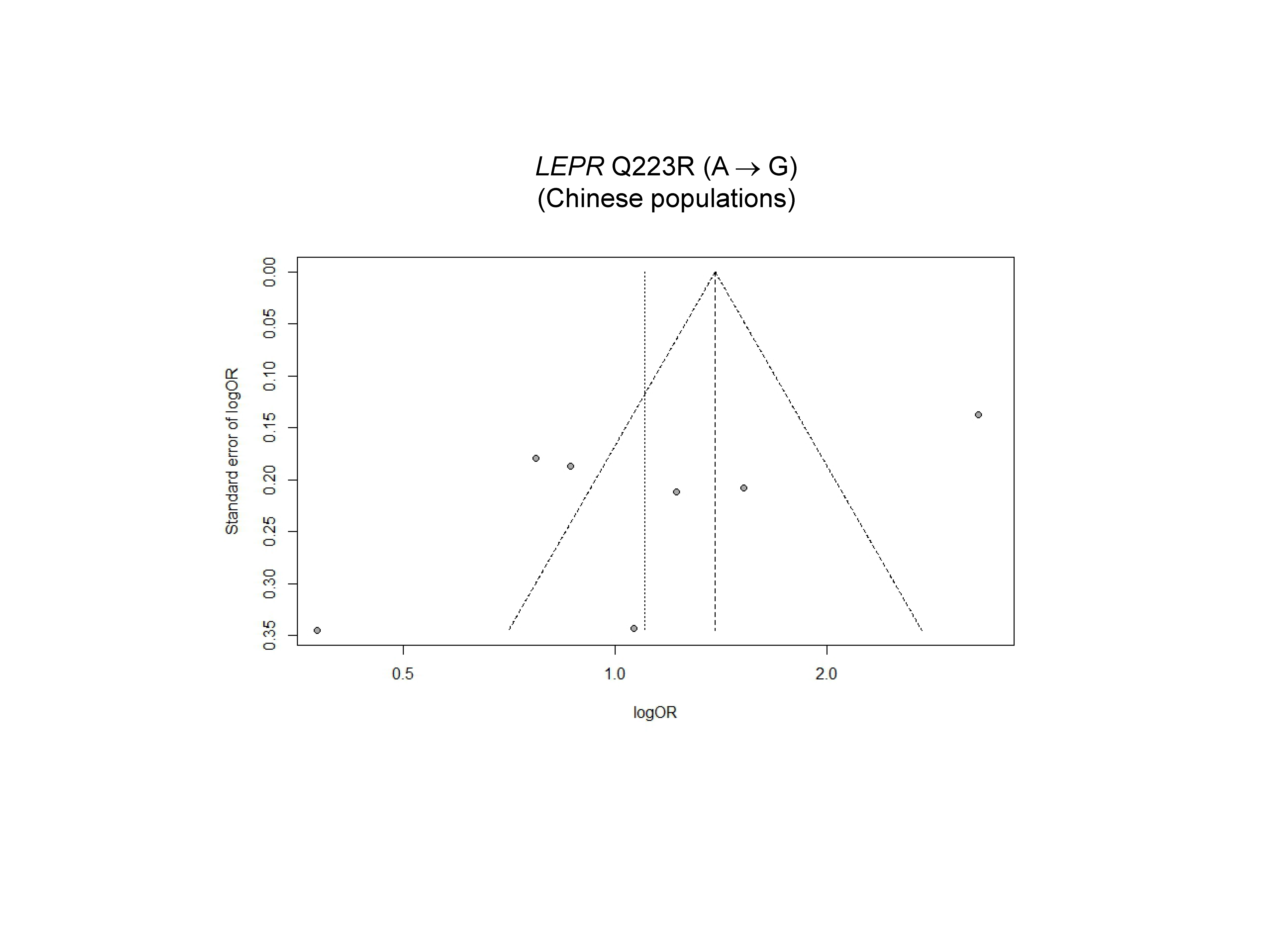

Supplement: S7 Fig — (TIFF) [file pone.0189366.s010.tiff]

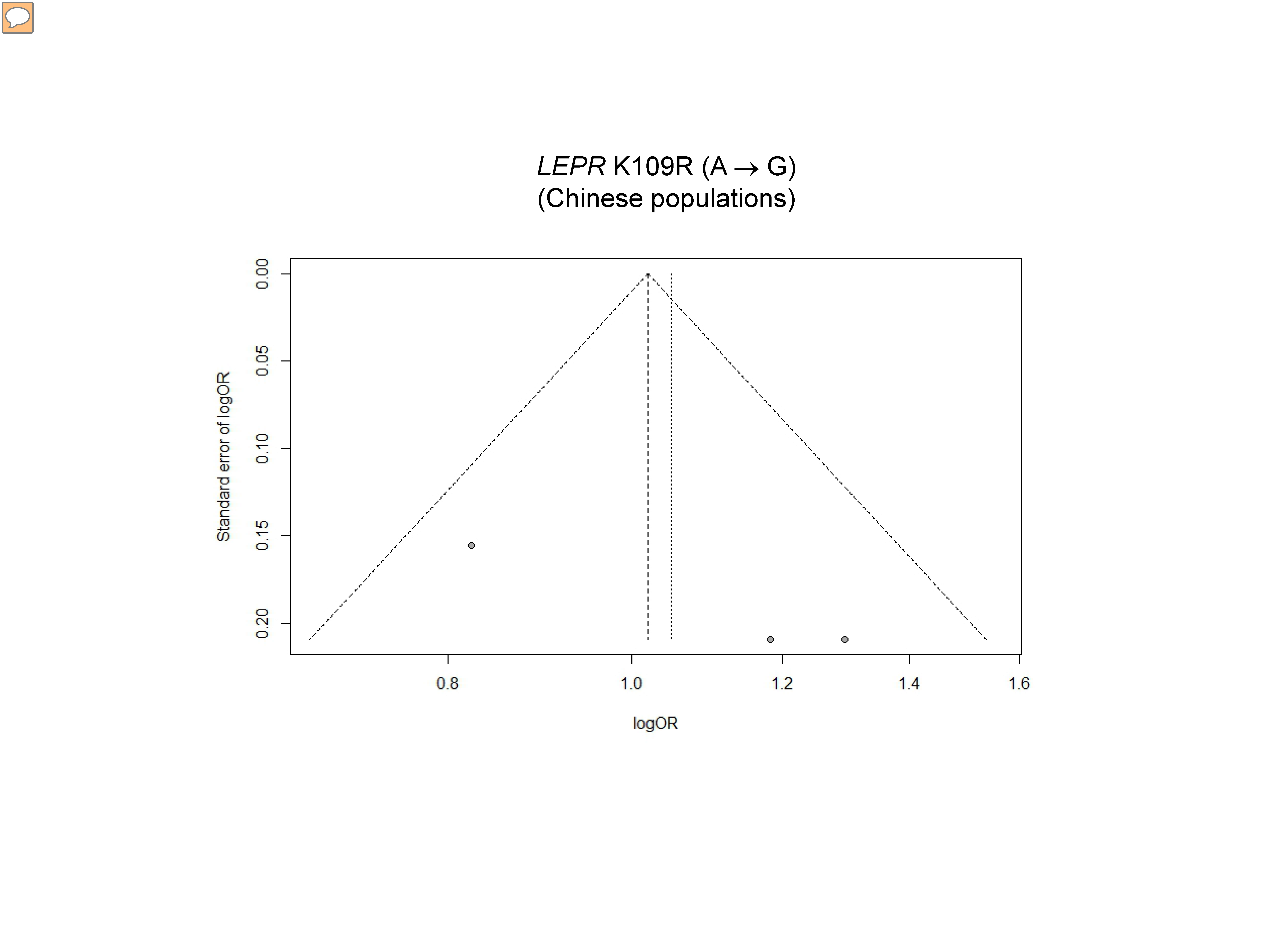

Supplement: S8 Fig — (TIFF) [file pone.0189366.s011.tiff]

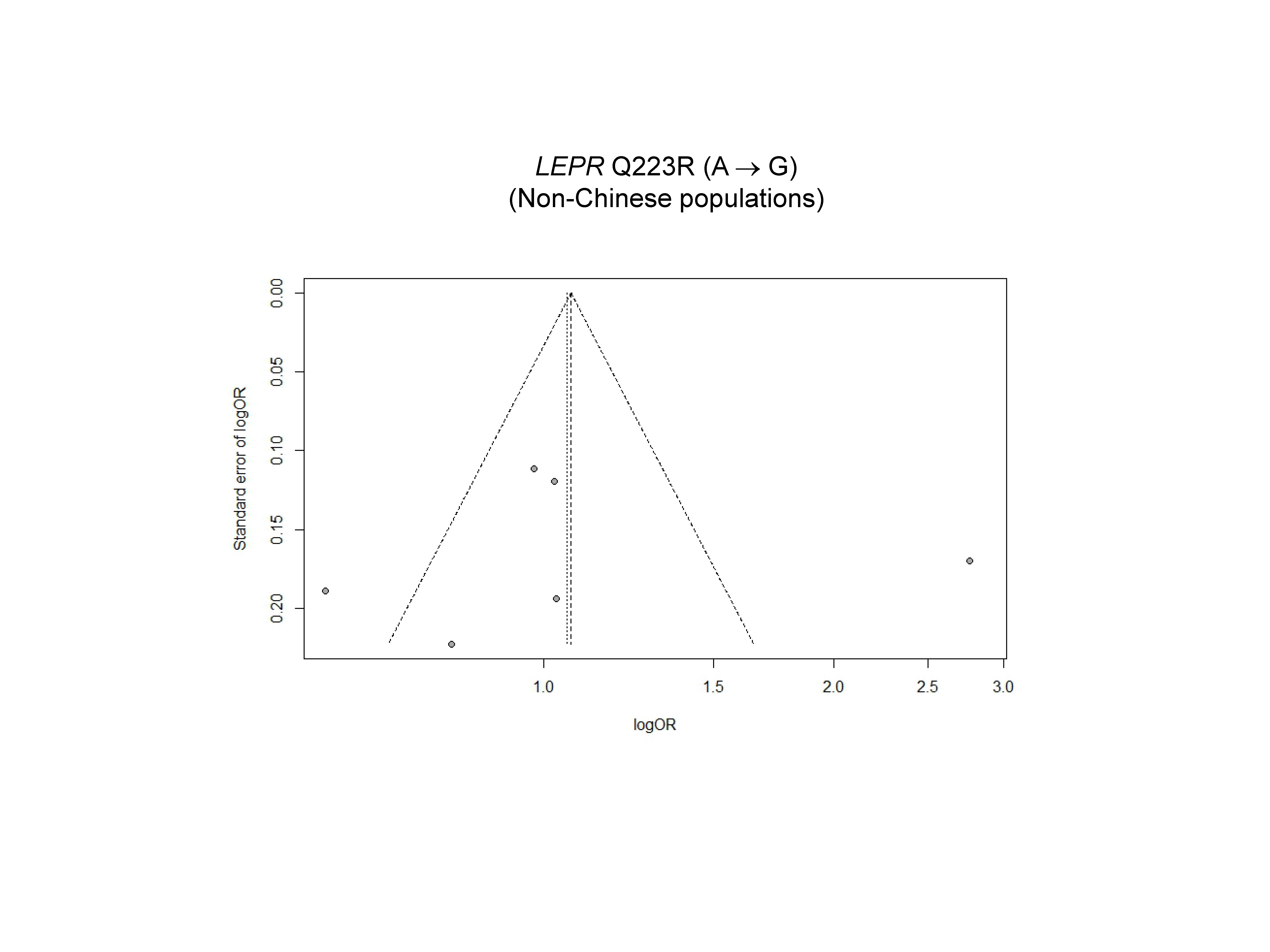

Supplement: S9 Fig — (TIFF) [file pone.0189366.s012.tiff]

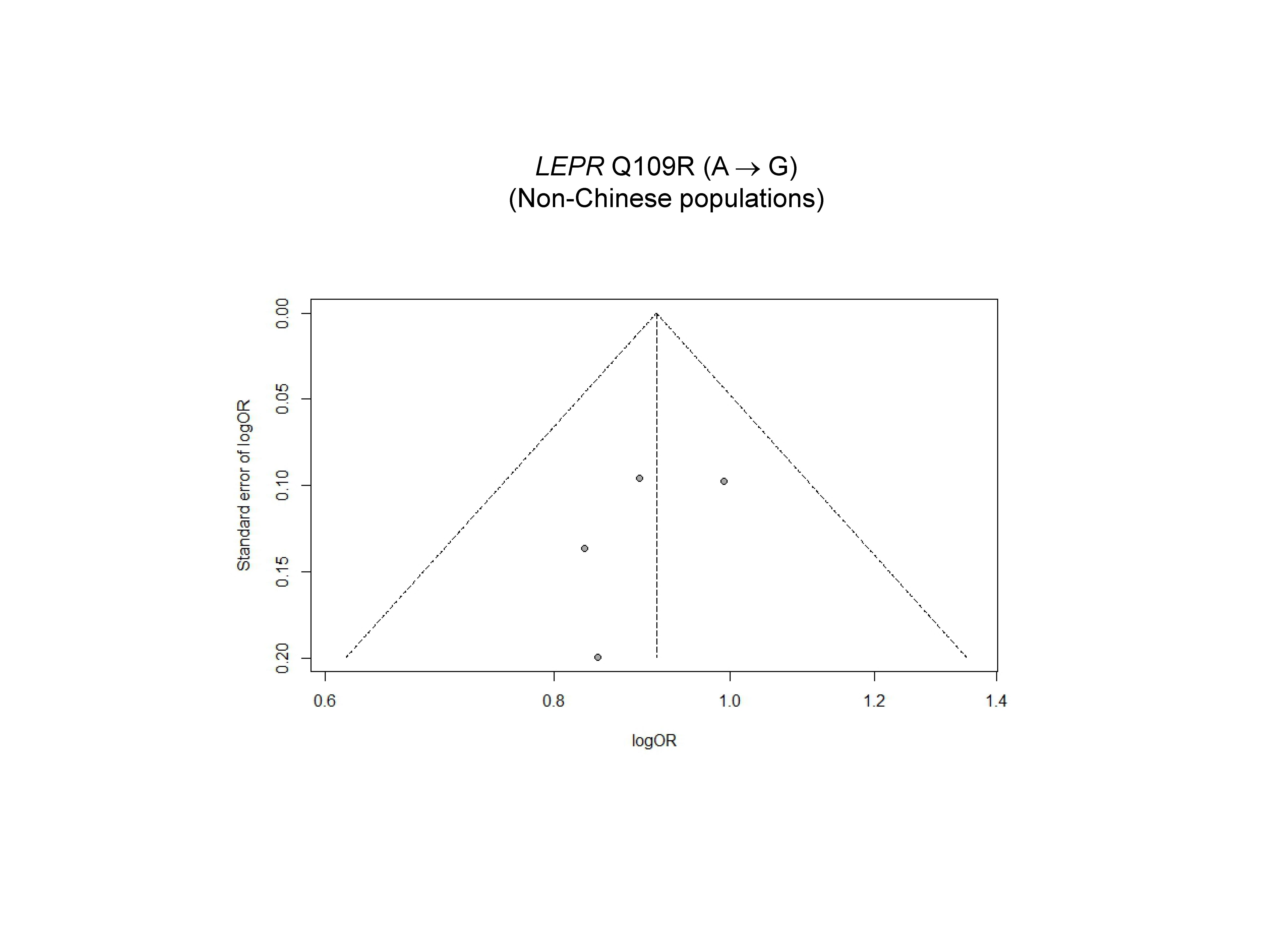

Supplement: S10 Fig — (TIFF) [file pone.0189366.s013.tiff]
